# Correcting gene expression data when neither the unwanted variation nor the factor of interest are observed

**DOI:** 10.1093/biostatistics/kxv026

**Published:** 2015-08-17

**Authors:** Laurent Jacob, Johann A. Gagnon-Bartsch, Terence P. Speed

**Affiliations:** Laboratoire de Biométrie et Biologie Évolutive, Université de Lyon, Université Lyon 1, CNRS, UMR, 5558 Lyon, France; Department of Statistics, University of California, Berkeley, CA 974720, USA; Department of Statistics, University of California, Berkeley, CA 974720, USA and Division of Bioinformatics, Walter and Eliza Hall Institute of Medical Research, Melbourne 3052, Australia

**Keywords:** Batch effect, Control genes, Gene expression, Normalization, Replicate samples

## Abstract

When dealing with large scale gene expression studies, observations are commonly contaminated by sources of unwanted variation such as platforms or batches. Not taking this unwanted variation into account when analyzing the data can lead to spurious associations and to missing important signals. When the analysis is unsupervised, e.g. when the goal is to cluster the samples or to build a corrected version of the dataset—as opposed to the study of an observed factor of interest—taking unwanted variation into account can become a difficult task. The factors driving unwanted variation may be correlated with the unobserved factor of interest, so that correcting for the former can remove the latter if not done carefully. We show how negative control genes and replicate samples can be used to estimate unwanted variation in gene expression, and discuss how this information can be used to correct the expression data. The proposed methods are then evaluated on synthetic data and three gene expression datasets. They generally manage to remove unwanted variation without losing the signal of interest and compare favorably to state-of-the-art corrections. All proposed methods are implemented in the bioconductor package RUVnormalize.

## 1. Introduction

Over the last few years, microarray-based gene expression studies involving a large number of samples have been conducted (Cancer Genome Atlas Research Network, 2008), with the goal of helping understand or predict some particular *factors of interest* like the prognosis or the subtypes of a cancer. Such large gene expression studies are often carried out over several years, may involve several hospitals or research centers and typically contain some *unwanted variation*. Sources of unwanted variation can be technical elements such as batches, different platforms or laboratories, or any biological signal which is not the factor of interest of the study such as heterogeneity in ages or different ethnic groups.

Unwanted variation can easily lead to spurious associations. For example when one is looking for genes which are differentially expressed between two subtypes of cancer, the observed differential expression of some genes could actually be caused by differences between laboratories if laboratories are partially confounded with subtypes. When doing clustering to identify new subgroups of the disease, one may actually identify some of the unwanted factors if their effects on gene expression are stronger than the subgroup effect. If one is interested in predicting prognosis, one may actually end up predicting whether the sample was collected at the beginning or at the end of the study because better prognosis patients were accepted at the end of the study. In this case, the classifier obtained would have little value for predicting the prognosis of new patients.

Similar problems arise when trying to combine several smaller studies rather than working on one large heterogeneous study: in a dataset resulting from the merging of several studies the strongest effect one can observe is generally related to the membership of samples to different studies. A very important objective is therefore to remove this unwanted variation without losing the variation of interest.

A large number of methods have been proposed to tackle this problem, mostly using linear models. When both the factor of interest and the unwanted factors are observed, the problem essentially boils down to a linear regression. ComBat (Johnson *and others*, 2007) is an empirical Bayes version of linear regression that has been shown to be quite effective. When the factor of interest is observed but the unwanted factors are not, the latter need to be estimated before a regression is possible. This can be done using some form of factor analysis (Leek and Storey, 2007, 2008; Gagnon-Bartsch and Speed, 2012), or using the entire covariance structure of the gene expression matrix (Kang *and others*, 2008; Listgarten *and others*, 2010). Finally if the factor of interest itself is not defined, some methods (Alter *and others*, 2000) use singular value decomposition (SVD) on gene expression to identify and remove the unwanted variation and others (Benito *and others*, 2004) remove observed batches by linear regression. A more detailed overview of the literature on this subject is provided in Section 1 of the Supplementary Material.

In this paper, we focus on this latter case where there is no predefined factor of interest. This situation arises when performing unsupervised estimation tasks such as clustering or principal component analysis (PCA), in the presence of unwanted variation. It can also be the case that one needs to normalize a dataset without knowing which factors of interest will be studied. Our objective is to correct the gene expression by estimating and removing the unwanted variation, without removing the—unobserved—variation of interest.

The recent work of Gagnon-Bartsch and Speed (2012) suggests that negative control genes can be used to estimate unwanted factors. Here we propose ways to improve estimation of the effect of these sources when the factor of interest is not observed. Our contributions here are 3-fold. We propose estimators which, given the unwanted factors estimated by Gagnon-Bartsch and Speed (2012), are well suited to estimating their effect in the presence of unobserved factors of interest. We introduce a different estimator which relies on replicate samples. Finally, we systematically compare existing and proposed correction methods on an extensive set of experiments.

Section 2 recalls the model of Gagnon-Bartsch and Speed (2012) and introduces estimators of the effect of a given unwanted factors, which are suited to the case where the factor of interest is unobserved. Section 3 describes an alternative estimator of the unwanted variation using replicate samples rather than the unwanted factors previously estimated using control genes. Section 4 compares existing and proposed correction methods on synthetic data, Section 5 does the same thing on a gene expression dataset.

## 2. Correction using negative control genes

The removal of unwanted variation (RUV) model used by Gagnon-Bartsch and Speed (2012) is a linear model first introduced in this context by Leek and Storey (2007), with a term representing the variation of interest and another term representing the unwanted variation:

(2.1) \begin{equation*} Y = X\beta + W\alpha + \varepsilon, \end{equation*}

with $Y \in \mathbb {R}^{m\times n}$, $X\in \mathbb {R}^{m\times
            p}$, $\beta \in \mathbb {R}^{p\times n}$, $W\in \mathbb {R}^{m\times
            k}$, $\alpha \in \mathbb {R}^{k\times n}$, and $\varepsilon \in \mathbb
            {R}^{m\times n}$. $Y$ is the observed matrix of expression of $n$ genes for $m$ samples, $X$ represents the $p$ factors of interest, $W$ the $k$ unwanted factors and $\varepsilon $ some noise, typically $\varepsilon
            _j \sim \mathcal {N}(0,\sigma ^2_\varepsilon I_m),\ j=1,\ldots
          ,n$. While Leek and Storey (2007) and Gagnon-Bartsch and Speed (2012) use a gene-specific variance $\sigma ^2_{\varepsilon _j}$, we restrict ourselves to a common variance in this work—Sections $3.7.4$ and $\textrm
          {A}.3$ in Gagnon-Bartsch *and others* (2013) provide a detailed and illustrated discussion of why this approximation is reasonable. Both $\alpha $ and $\beta $ are modeled as fixed, i.e., non-random.

Gagnon-Bartsch and Speed (2012) were mainly interested in the case where $X$ is an observed factor of interest, and the objective is to test which genes are affected by this factor of interest—whether $\beta _j =
          0$ for each gene $j$. If $W$ is also observed, the maximum likelihood estimator of $\beta $ is a well studied linear regression estimator. The major contribution of Gagnon-Bartsch and Speed (2012) is to provide an estimator of $W$ exploiting the fact that some genes are known to be negative controls, i.e., unrelated to the factor of interest $X$. We refer to this estimator as $\hat {W}_{2}$ in the remaining of this article. By contrast in this work, we are interested in the case where $X$ is not observed. Our objective in general will be to estimate $W\alpha
          $ and remove it from $Y$.

### 2.1 A random effect version of RUV for unobserved $X$

If $X$ is not observed, $(X\beta , \alpha )$ become non-identifiable even given $W$. A naive solution is to estimate $\alpha
            $ by regression of $Y$ on $W$, i.e., to project $Y$ onto the orthogonal complement of $W$. This approach is referred to as *naive RUV-2* in Gagnon-Bartsch and Speed (2012) and is expected to be helpful as long as $X$ and $W$ are not too correlated. If however there is some degree of confounding between the factor of interest and the unwanted variation sources, such a radical removal of the latter will remove too much of the former. In an extreme example, if one studies the effect of a treatment on gene expression and all treated samples are done on Day 1 and all untreated samples on Day 2, removing all variation along the Day 1–Day 2 axis also removes all variation between treated and untreated samples.

We now discuss how a random $\alpha $ version of (2.1) could improve estimation when $X$ and $W$ are not expected to be orthogonal.

The naive RUV-2 estimator of $\alpha $ is formally given by

(2.2) \begin{equation*} \min_{\alpha\in\mathbb{R}^{k\times n}} \|Y - \hat{W}_{2} \alpha\|^2_F, \end{equation*}

which is the maximum likelihood estimator of $\alpha
              $ for model (2.1) if $W=\hat
              {W}_{2}$ and $X\beta =0$. If we keep the same model and endow $\alpha
              $ with a distribution $\alpha _j \stackrel {iid}{\sim } \mathcal
              {N}(0,\sigma _\alpha ^2I_k),\ j=1,\ldots ,n$, the maximum a posteriori estimator of $\alpha $ becomes:

(2.3) \begin{equation*} \min_{\alpha\in\mathbb{R}^{k\times n}} \{\|Y - \hat{W}_{2} \alpha\|^2_F + \nu \|\alpha\|^2_F\}, \end{equation*}

where $\nu =\sigma _\varepsilon
              ^2/\sigma _\alpha ^2$. Here again like with $\sigma _\varepsilon
              $, we limit ourselves to a model where $\sigma _\alpha
              $ is common to all genes. Sections 14 and 15 of the Supplementary Material discuss a related model where $W$ rather than $\alpha $ is modeled as a random quantity.

The only difference with the naive RUV-2 estimator (2.2) is the $\ell _2$ penalty term: (2.3) is a ridge regression against $\hat
            {W}_{2}$ whereas (2.2) is an ordinary regression. In this context where $X$ is unobserved and $X\beta $ is set to 0 to estimate $\alpha
              $, this difference can be important if $X$ and $W$ are correlated—for more detail, see Section 3 of the Supplementary Material.

### 2.2 Generalization: joint estimation of $X\beta $ and $\alpha $

Assuming some structure is known on the unobserved $X\beta $ term, it is possible to write a joint estimator of $(X\beta , \alpha )$ given $W$ rather than fixing $X\beta =0$:

(2.4) \begin{equation*} \min_{X\beta \in \mathcal{M}} \min_{\alpha\in\mathbb{R}^{k\times n}} \{\|Y - W\alpha - X\beta\|^2_F + \nu \|\alpha\|^2_F\}, \end{equation*}

where $\mathcal
            {M}$ is a subset of $\mathbb {R}^{m\times
            n}$. A typical example of $\mathcal {M}$ would be a clustering structure $\{X\beta ~: X\in ~\mathcal {C}, \beta \in \mathbb {R}^{k\times
            n}\}$, where $\mathcal {C}$ denotes the set of cluster membership matrices $\mathcal
              {C} \stackrel {\Delta }{=} \{M \in \{0,1\}^{m\times k}, \sum _{j=1}^k M_{i,j} = 1,
              i=1,\ldots ,m \}$. In this case, the minimization over $X\beta
            $ in (2.4) for a given $W\alpha $ can be addressed by a *k*-means algorithm over $Y - W\alpha
              $. Other typical examples include PCA where $\mathcal {M}=\{M
              :\textrm {rank}(M)\leq p\}$ and sparse dictionary learning (Mairal *and others*, 2010) where $\mathcal {M}=\{X\beta
              :\textrm {rank}(X\beta )\leq p, \|X_i\|\leq 1, i=1,\ldots ,p, \|\beta \|_1 \leq \mu
              \}$. Section 12 of the Supplementary Material provides an alternative formulation of (2.4).

The objective of this joint modeling can be 2-fold: one may still just want to estimate $\alpha
            $ in order to return a corrected $Y - W\alpha $ matrix, but hope that the joint estimation will yield a better estimate of $\alpha
            $ (in the sense of $\|\hat {\alpha } - \alpha
            \|^2$). One may also be actually interested in estimating the unobserved $X\beta
              $, e.g., to solve a clustering problem in the presence of unwanted variation.

A joint solution for $(X\beta ,\alpha )$ is generally not available for (2.4). A possible way of maximizing the likelihood of $X\beta
              $ however is to alternate between a step of optimization over $\alpha
            $ for a given $X\beta $, which corresponds to a ridge regression problem, and a step of optimization over $X\beta $ for a given $\alpha
            $ using the relevant unsupervised estimation procedure over $Y - W\alpha
            $. Each step decreases the objective $\|Y - X\beta - W\alpha
              \|_F + \nu \|\alpha \|_F^2$, and even if this procedure does not converge in general to the global maximum likelihood of $(X\beta , \alpha )$, it may yield better estimates than (2.3) where $X\beta
              $ is simply assumed to be 0.

Finally, such a joint scheme can be used to build a different estimator of $W$, akin to the feasible generalized least squares (Freedman, 2005) used in regression: once an estimate of $X\beta |W,
              \alpha $ becomes available, $W$ can be re-estimated using and SVD on the residuals $Y - \hat {X\beta }$ rather than the control genes. This approach is discussed in Section 2 of the Supplementary Material.

## 3. Correction using replicate samples

We now introduce an alternative estimator of the unwanted variation $W\alpha
          $, which, unlike the ones discussed in Section 2 does not rely on a previous estimator of $W$. Symmetrically to the negative control genes used to estimate $W$ in Section 2, we now consider negative “control samples” for which the factor of interest $X$ is 0.

In practice, one way of obtaining such control samples is to use replicate samples, i.e., samples that come from the same tissue but which were hybridized in two different settings, say across time or platform. The profile formed by the difference of two such replicates should only be influenced by unwanted variation—those whose levels differ between the two replicates. In particular, the $X$ of this difference should be 0. By construction, this approach is only able to deal with unwanted variation with respect to which replicates are available, which is often the case for technical unwanted variation but rarely the case for biological unwanted variation.

More generally when there are more than two replicates, one may take all pairwise differences or the differences between each replicate and the average of the other replicates. We will denote by $d$ the indices of these artificial control samples formed by differences of replicates, and we therefore have $X^d = 0$ where $X^d$ are the rows of $X$ indexed by $d$.

Such samples can then be used to estimate $\alpha $ in a way that is dual to the way (Gagnon-Bartsch and Speed, 2012) used control genes to estimate $W$, recalled in Section 3 of the Supplementary Material. More precisely, we consider the following algorithm:

- Use the rows of $Y$ corresponding to control samples $Y^d = W^d\alpha +
                \varepsilon ^d$ to estimate $\alpha $. Assuming i.i.d. noise $\varepsilon
                _j \sim \mathcal {N}(0,\sigma ^2_\varepsilon I_m),\ j=1,\ldots
              ,n$, the $(W^d\alpha )$ matrix maximizing the likelihood of this model is ${\rm argmin}_{W^d\alpha ,\,\textrm {rank}\,W^d\alpha \geq k}\|Y^d
                -W^d\alpha \|_F^2$. By the same argument used to compute $\hat
                {W}_{2}$, this argmin is reached for $\widehat {W^d\alpha }
                = PE_kQ^\top $, where $Y^d = PEQ^\top $ is the SVD of $Y^d$ and $E_k$ is the diagonal matrix with the $k$ largest singular values as its $k$ first entries and 0 on the rest of the diagonal. We can use $\hat {\alpha } =
                E_k Q^\top $.
- Using $\hat {\alpha
                }$ in the restriction $Y_c = W\alpha _c + \varepsilon
              _c$ of $Y$ to the control gene columns, the maximum likelihood estimate of $W$ is now solved by a linear regression, $\hat
                {W}_{r}\stackrel {\Delta }{=} Y_c \hat {\alpha }^\top _c (\hat {\alpha }_c\hat
                {\alpha }_c^\top )^{-1}$.
- Once $W$ and $\alpha $ are estimated, $\hat {W}\hat {\alpha
                }$ can be removed from $Y$.

$X$ is not required in this procedure which constitutes a fully unsupervised correction for $Y$.

The extreme case where all genes are used as control genes is of interest, and is discussed in Section 10 of the Supplementary Material.

Finally, this replicate-based correction yields an estimator $\hat {W}_{r}$ of $W$, obtained by regression of $Y_c$ against $\hat {\alpha
            }_c$. This estimator could be used in any of the methods described in Section 2. Section 11 of the Supplementary Material discusses the difference between $\hat
            {W}_{r}$ and $\hat {W}_{2}$.

## 4. Result on synthetic data

We start with a set of experiments on synthetic data, where we generate the data ourselves. In this case, we are able to measure how well each correction method recovers the true matrix $Y - W\alpha
            $ and it makes sense to talk about “right” or “best” corrections.

### 4.1 Protocol

We generate data according to model (2.1). $X$ has two columns: one is a binary variable, the other an associated continuous variable. One can think of the binary variable as some clinical grouping of the tumor, such as the ER status for breast cancer. The continuous variable could be survival time. More precisely the survival covariate is sampled from an exponential law with parameter $0.05$ for ER$+$ samples and $0.1$ for ER$-$ samples. $W$ also has two columns, a binary one which could be the technical platform, and a continuous one which could be the RNA quality. RNA quality is sampled from a normal distribution—independently from the technical platform.

The columns of $X$ and $W$ are then transformed to have norm 1. We generate $m=100$ samples with $n=10\,000$ genes each using model (2.1). The $\alpha
              _{ij}$ and $\beta _{ij}$ parameters are iid sampled from a normal distribution with mean 0 and variance 1, except for the 100 control genes, for which $\beta
              _{ij}=0$. The $\varepsilon _{ij}$ parameters are i.i.d. sampled from a normal distribution with mean 0 and variance $0.01$. We also generate 10 additional samples with 2 replicates each, with the same values of $X$ but different values of $W$.

We compare the performances of ComBat (Johnson *and others*, 2007), quantile normalization (QN, Bolstad *and others*, 2003), naive RUV-2, the random effect model above, and the replicate-based model on simulated data. ComBat cannot deal with continuous unwanted variation factors and is only given the binary unwanted one. Each method using negative control genes is given either the actual negative control genes, or everything but the negative control genes (“poor control genes” version). Furthermore, we try two iterative methods, as described in 2.2. We perform 100 iterations: in the first version, $W$ is estimated once and for all from control genes like in the non-iterative random effect estimator, and in the second version it is re-estimated from the $Y-\widehat {X\beta
              }$ as detailed in Section 2 of the Supplementary Material after every 34 iterations.

Table 1 shows the performances of each correction method in three different settings: canonical correlations (Hotelling, 1936) between $X$ and $W$ equal to $(0.13, 0.05)$ (*Independent*), $(1, 1)$ (*Confounded*), and $(0.99,
            0.8)$ (*Moderate*). The normalized reconstruction error is measured by $\|(Y - \widehat {W\alpha }) - (Y- W\alpha )\|^2/\|Y - W\alpha
              \|^2$: our objective is to remove the unwanted variation from $Y$, and only the unwanted variation. These performances are those obtained when using the hyperparameter used to generate the data for each method. The effect of misspecification for $k$ and $\nu $ is discussed in Section 8 of the Supplementary Material. 

**Table 1.** Simulations: reconstruction error after various corrections in the three studies settings

	Independent	Confounded	Moderate
Uncorrected	0.67	0.68	0.67
QN	0.74	0.66	0.63
ComBat	0.34	0.66	0.76
naive RUV2	0.17	0.67	0.53
naive RUV2 poor control	0.74	0.67	0.67
random effect	0.23	0.34	0.31
random poor control	0.48	0.37	0.38
Iterative	0.11	0.46	0.19
Iterative update	0.06	0.40	0.16
Replicates	0.32	0.30	0.30
Replicates poor control	0.28	0.28	0.28

### 4.2 Result

In the case, where $X$ and $W$ are generated independently, the QN-corrected matrix yields a larger reconstruction error than the uncorrected one. ComBat helps, because it removes all of the platform effect without affecting much the signal along $X$. Naive RUV-2 gets even better results because it also accounts for the continuous unwanted factor, but its performance is severely reduced if we use non-control genes: in this case, the estimated $W$—by PCA on the non-control genes—is associated with the true $X$, which leads to removing too much variance along $X$.

The random effect model yields similar performances as naive RUV-2, slightly worse because it uses $k=m$ and therefore shrinks the signal in every direction. It is also affected by the use of non-control genes, but less dramatically than naive RUV-2 because it does not remove all the signal along the estimated $W$. The iterative methods greatly improves the performances.

The replicate-based method obtains a performance similar to that of ComBat. Interestingly, its results are slightly improved by using non-control genes, see Section 10 of the Supplementary Material for a detailed discussion of this phenomenon.

When $X=W$ (*Confounded*), QN, ComBat and naive RUV-2 lead to the same reconstruction error as the uncorrected matrix. They actually fail for opposite reasons: the unwanted variation along $W=X$ adds variance along $X$, so the uncorrected matrix has too much variance along this direction. By contrast, ComBat/naive RUV-2 remove too much variance along $X=W$, because they treat all of it as unwanted variation. The random effect method performs a bit less well than in the independent case, but is not as dramatically affected by the confounding as the fixed effect naive RUV-2 method. Importantly, the iterative methods decrease the performance with respect to the non-iterative random effect estimator. The iterative methods only help if they manage to estimate $X\beta
              $ well enough to improve the estimation of $W\alpha
            $. In this setting, $X=W$ so removing $\widehat {W\alpha
              }$ decreases the variance along $X$ too much, and makes it harder to identify it properly.

Finally, as expected from the discussion of Section 11 of Supplementary Material, the replicate-based method is not affected by the confounding at all: by construction, it only considers the variance coming from the technical unwanted variation.

The last column illustrates an intermediate case with moderate confounding. Random effect still works much better than uncorrected, QN, ComBat and naive RUV-2, and is less affected by the use of non-control genes. It is worth noting that even if $X$ and $W$ are highly correlated, the iterative methods yield a much lower error than the non-iterative one, suggesting that they only reduce performances in extreme cases like the total confounding setting.

We now summarize our observations on these synthetic experiments. ComBat performs well to remove an observed batch if it is largely independent from the signal of interest. Naive RUV-2 performs well to remove a batch—observed or not—if it is given good control genes and if the batch is not too associated with the factor of interest. The random effect model performs like naive RUV-2 but is much less affected by confounding and poor control genes. Iterating the estimation of $\alpha $ given $(W, X\beta )$ and $X\beta
            $ given $W\alpha $ improves the estimate of $W\alpha
            $, even more so if the residuals $Y - X\beta $ are used to update the estimate $W$. This strategy fails however when $X\beta
            $ is too hard to estimate, in which case iterations can even reduce the performances. Finally, the replicate-based method is not affected at all by control gene quality or confounding, it only depends on the number/coverage of replicates—see Sections 10 and 13 of the Supplementary Material for additional discussion of these points.

## 5. Result on real data

On real data, we have no way to measure how close we are to the true $Y - W\alpha
          $ matrix. As a surrogate, we choose datasets for which we know a factor of interest $X$, and measure how well this factor is recovered by clustering on each corrected gene expression matrix. The correction methods are not allowed to use the known groups, to emulate a problem where the factor of interest is not observed. This surrogate is admittedly imperfect, as other factors of interest may be present in these datasets. Whether or not removing the effect of these (possibly biological) other factors is desirable depends on whether they are considered “wanted” or “unwanted” in any particular analysis. We consider these surrogates to be complementary to the synthetic datasets of Section 4—which are not real data but where we can define what the right correction is.

This section presents results on the microarray gene expression datasets studied in Gagnon-Bartsch and Speed (2012). We use the gender partition as the factor of interest to be recovered, implicitly treating other factors as unwanted variation, but discuss other options in Section $4.3$ of the Supplementary Material. Two additional datasets are studied in Sections 5 and 6 of the Supplementary Material, and Section 7 of the Supplementary Material shows the importance of having good negative control genes on correction quality.

### 5.1 Protocol

For each of the correction methods that we evaluate, we apply the correction method to the expression matrix $Y$ and then estimate the clustering using a $k$-means algorithm. To quantify how close each clustering gets to the objective partition, we adopt the following squared distance between two given partitions $\mathcal
              {C}=(c_1,\ldots ,c_k)$ and $\mathcal {C}'=(c'_1,\ldots
            ,c'_k)$ of the samples into $k$ clusters: $d(\mathcal {C},\mathcal
              {C}') = k - \sum _{i,j=1}^k ({|c_i \cap
            c'_j|^2}/{|c_i||c'_j|})$, where $|S|$ denotes the cardinal of a set $S$. This score ranges between 0 when the two partitions are equivalent, and $k-1$ when the two partitions are completely different. To give a visual impression of the effect of the corrections on the data, we also plot the data in the space spanned by the first two principal components.

We evaluate two basic correction methods: the replicate-based procedure described in Section 3 and the random $\alpha $ model of Section 2.1 with $\hat {W} = \hat
            {W}_{2}$. For each of these two methods, we also evaluate iterative versions as described in Section 2.2. $X\beta |W\alpha
              $ is estimated using the sparse dictionary estimator of Mairal *and others* (2010), which minimizes $1/2
              \|(Y-\widehat {W\alpha }) - X\beta \|_F^2 + \lambda \|\beta
            \|_1$ under the constraint that $X\beta $ has rank $p$ and the columns of $X$ have norm 1. $W$ is re-estimated using the $Y-\widehat {X\beta }$ residuals every 10 iterations as discussed in Section 2 of the Supplementary Material. In addition, we consider as baselines (i) an absence of correction, (ii) a centering of the data by level of the known unwanted factors—similar to the correction provided by ComBat, and (iii) the naive RUV-2 procedure.

Some of the methods we compare require the user to choose some hyperparameters: the ranks $k$ of $W\alpha $ and $p$ of $X\beta
            $, the ridge parameter $\nu $ and the strength $\lambda
              $ of the $\ell _1$ penalty on $\beta
            $. On synthetic data, it makes sense to define which hyperparameters yield the best correction. On real data by contrast, different choices of these hyperparameters may lead to throwing away or keeping different signals, which can be a good or a bad thing depending on what downstream analysis is decided afterward. This point is illustrated in Section $4.3$ of the Supplementary Material: large values of $\nu
            $ lead to a clustering by brain region while smaller values lead to a clustering by gender.

No single rule can be therefore given as to hyperparameter choice and judgment is necessary each time adjustments are performed without a specified factor of interest. We suggest using relative log expression (RLE) plots, clustering with respect to a known factor of interest, or known differentially expressed genes with respect to a known factor of interest as positive controls. In this experiment, we compare normalization methods based on how well they allow clustering to recover the gender partition, so it would not make sense to use the same criterion to choose $\nu $. In Section 4 of the Supplementary Material, we use RLE plots to pick $\nu
              $ for this dataset, and also discuss the other criteria (see Table 1 of the Supplementary Material).

Since $\nu
            $ acts on the eigenvalues of $W^\top W$, we recommend considering a grid of powers of 10 of the largest of them—the discussion in the Supplementary Material regards how to choose the power. The rank $k$ of $W\alpha $ was chosen to be close to $m/4$, or to the number of replicate samples when the latter was smaller than the former. For methods using $p$, we chose $p=k$. For random $\alpha $ models, we use $k=m$: the model is regularized by the ridge $\nu
            $ and we do not combine it with a regularization of the rank. Finally, in order to make iterative and non-iterative methods comparable, we choose $\lambda
            $ for each method such that $\|W\alpha \|_F$ is close to the one obtained with its non-iterative counterpart.

### 5.2 Result

Vawter *and others* (2004) studied differences in gene expression between male and female patients. Gagnon-Bartsch and Speed (2012) used the resulting dataset to study the performances of RUV-2.

This gender study is an interesting benchmark for methods aiming at removing unwanted variation as it expected to be affected by several technical and biological factors: two microarray platforms, three different labs, three tissue localizations in the brain. Most of the 10 patients involved in the study had samples taken from the anterior cingulate cortex (a), the dorsolateral prefontal cortex (d), and the cerebellar hemisphere (c). Most of these samples were sent to three independent labs: UC Irvine (I), UC Davis (D) and University of Michigan, Ann Arbor (M).

Gene expression was measured using either HGU-95A or HGU-95Av2 Affymetrix arrays with 12 600 genes shared between the two platforms. Six of the $10\times 3\times 3$ combinations were missing, leading to 84 samples. We use as control genes the same 799 housekeeping probesets, which were used in Gagnon-Bartsch and Speed (2012). The proportion of genes on the sex chromosomes is similar in the housekeeping genes ($3\%$) and other genes ($4\%$).

For the replicate-based method of Section 3, we use all possible pairs that either differ in lab, but are otherwise identical in terms of chip type, the patient, and brain region, or (ii) differ in brain region but are otherwise identical in terms of chip type, the patient, and lab, leading to 106 differences. Finally as a pre-processing, we also mean-center the samples per array type.

Since most genes function irrespective of gender, clustering by gender gives better results in general when removing genes with low variance before clustering. For each method, we therefore apply clustering after filtering different numbers of genes based on their variance after correction.

**Fig. 1.**
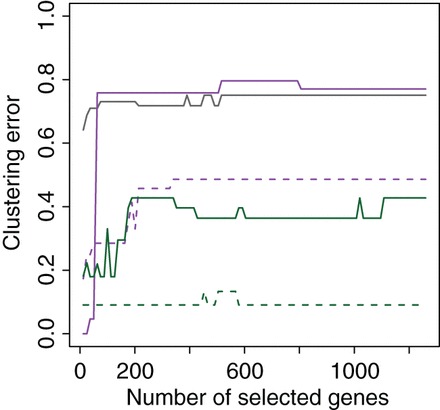
Clustering error against number of genes selected (based on variance) before clustering. From top to bottom at 1260 genes: replicate-based correction (full purple); naive RUV-2 (full gray); iterated replicate-based correction (dashed purple); random $\alpha
                  $ model using $\hat {W}_2$ (full green); iterated random $\alpha $ model using $\hat {W}_2$ (dashed green).

**Fig. 2.**
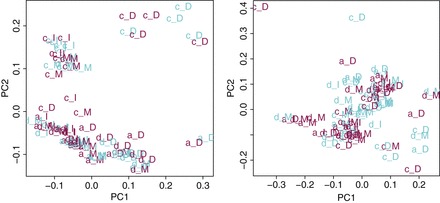
Samples of the gender study represented in the space of their first two principal components before correction (left panel) and after centering by lab plus brain region (right panel). Light blue samples are males, dark pink samples are females. The labels indicate the laboratory and brain region of each sample. The capital letter is the laboratory and the lowercase one is the brain region.

Figure 1 shows the clustering error for the methods against the number of genes retained. The uncorrected and mean-centering cases are not displayed to avoid cluttering the plot, but give values above $0.95$ for all numbers of genes retained. Figure 2 shows the samples in the space of the first two principal components in these two cases, keeping the 1260 genes with highest variance. On the uncorrected data (left panel), it is clear that the samples first cluster by lab which is the main source of variance, then by brain region which is the second main source of variance. This explains why the clustering on uncorrected data is far away from a clustering by gender. Mean-centering samples by region-lab (right panel) removes all clustering per brain region or lab, but does not make the samples cluster by gender.

The gray line of Figure 1 shows the performance of naive RUV-2 for $k=20$. Since naive RUV-2 is a radical correction which removes all variance along some directions, it is expected to be more sensitive to the choice of $k$. The estimation is damaged by using $k=40$ (clustering error $0.99$). Using $k=5$ also degrades the performances, except when very few genes are kept.

The purple lines of Figure 1 represent the replicate-based corrections. The solid line shows the performances of the non-iterative method described in Section 3. When very few genes are selected, it leads to a perfect clustering by gender, which no other method achieves regardless of the number of genes they retain. When considering more genes, however, its performance become similar to the one of naive RUV-2, suggesting that additional genes are influenced by non-gender variation which the replicate-based method does not remove. It is expected that a few genes are more strongly affected by gender than the others, so it makes sense for a correction method to recover a better clustering by gender after restriction to a small number of high variance genes. In addition, Table 1 of the Supplementary Material shows that even though the replicate-based method has a large clustering error, it actually performs as well as or better than other methods in terms of number of differentially expressed genes on the sex chromosomes.

**Fig. 3.**
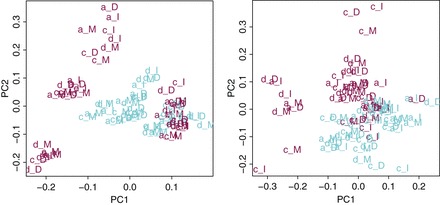
Using replicates. Left: no iteration and right: with iterations.

The iterative version in dotted line leads to much better clustering except again when very few genes are selected. Figure 3 shows the samples in the space of the first two principal components after applying the non-iterative (left panel) and iterative (right panel) replicate-based method. The correction shrinks the replicates together, leading to a new variance structure, more driven by gender although not separating perfectly males and females.

**Fig. 4.**
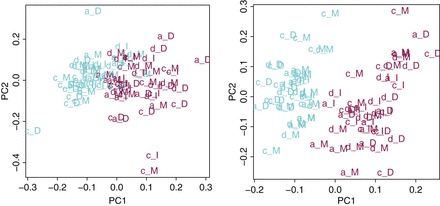
Random alpha with control genes only. Left: no iteration and right: with iterations.

The green lines of Figure 1 correspond to the random $\alpha
            $-based corrections. The solid line shows the results for the non-iterative method. These results are good, as illustrated by the reasonably good separation obtained in the space spanned by the first two principal components after correction on the left panel of Figure 4. The dotted green line corresponds to the random $\alpha $-based corrections with iterations plus sparsity, which leads to an even lower clustering error.

## 6. Discussion

We introduced methods to estimate and remove unobserved unwanted variation from gene expression data when the factor of interest is also unobserved. One method uses the negative control gene-based estimator of unwanted factors introduced in Gagnon-Bartsch and Speed (2012), and estimates the effect of these factors on gene expression using a random effect model. The second method relies on replicate samples and estimates the unwanted variation using the variation observed in differences of replicates. Both estimators can be improved by joint modeling of the variation of interest and the unwanted variation. All the methods we introduce are available in the bioconductor package RUVnormalize (Jacob, 2014).

We systematically compared the proposed correction techniques with state-of-the-art methods on both synthetic and real gene expression data. On synthetic data, we knew what the correct signal was, and could measure how well each correction method recovered this signal. When good control genes were available, the random effect estimator performed much better than existing correction methods in the presence of confounding. The replicate-based method performed less well than the control gene based one—unless a really large number of replicates was available—but was unaffected by poor quality control genes and to large confounding level. We were able to verify that both proposed methods provide a better correction even in the case where the factor of interest and the unwanted factors are totally confounded.

On real gene expression data where it did not make sense to define a single correct signal to be recovered, we assessed how well we were able to rediscover by clustering a known factor of interest which was unspecified at correction time. Here again, the proposed methods lead to better reconstruction than existing corrections.

Assessing how well each unsupervised correction method works on a new real dataset is problematic, since the factor of interest is not observed. Clustering with respect to a known biological factor, like we do with gender, is one option to perform this assessment. Other options include using positive control genes and RLE plots, like we do in the Supplementary Material. None of these options is perfect but they can be used as guidelines, to monitor whether too much variance is being removed by any correction method. In particular, they can and should be used to choose regularization parameters such as the rank $k$ of $\hat {W}$ and the ridge $\nu
          $ of random $\alpha $ approaches. In any case, one should keep in mind that optimizing for one known thing may not optimize for another: in our gender data example, the parameters which were chosen by RLE and behaved well for gender recovery are not optimal for recovering a partition by brain region.

To conclude, our results suggest that it is possible to remove unwanted variation from gene expression without losing the signal of interest, provided enough controls are available: negative control genes which are affected by the unwanted factors only, or replicate samples. Together with other researchers in our groups we have also started applying some of the methods that we introduce here to RNA-Seq (Risso *and others*, 2014), metabolomics (Livera *and others*, 2015) and expression array data (Jacob *and others*, 2015) and obtained consistently good results. We hope these extensive evaluations and comparisons will be helpful to future researchers trying to remove unwanted variation from their data.

## Supplementary material

Supplementary material is available at http://biostatistics.oxfordjournals.org.

## Funding

This work was funded by the SU2C-AACR-DT0409 grant. Funding to pay the Open Access publication charges for this article was provided by Australian National Health and Medical Research Council Program Grant APP1054618.

## Supplementary Material

kxv026_Supplementary_Material
