# Supplementary material for: Correcting gene expression data when neither the unwanted variation nor the factor of interest are observed
Source: Biostatistics. 2015 Aug 17;17(1):16–28. doi: 10.1093/biostatistics/kxv026 (PMC4679071; doi:10.1093/biostatistics/kxv026)
Supplement: kxv026_Supplementary_Material [file kxv026_supplementary_material.pdf]

Biostatistics (2017), **0**, 0, *pp.* 1–51
doi:10.1093/biostatistics/supplementary-final

Supplementary material to "Correcting gene expression data when neither the unwanted variation nor the factor of interest are observed"

LAURENT JACOB*[,1], JOHANN GAGNON-BARTSCH[2], TERENCE P. SPEED[2,3]
[1]*Université de Lyon; Université Lyon 1; CNRS; UMR 5558, Laboratoire de Biométrie et Biologie Évolutive, Lyon, France*
[2]*Department of Statistics, University of California, Berkeley, USA*
[3]*Division of Bioinformatics, Walter and Eliza Hall Institute of Medical Research, Melbourne, Australia*

laurent.jacob@univ-lyon1.fr

1. Related work

The difficulty of estimating unwanted variation depends on what is actually considered to be observed and what is not.

1.1 *When both the factor of interest and the unwanted factors are observed*

If both the factor of interest and all the sources of unwanted variation (say technical batches or different countries) are known and assuming a linear model, the problem boils down to a linear regression: the expression of each gene is decomposed as an effect of the factor of interest plus an effect of the unwanted factors. When the variance of each gene is assumed to be different within each batch, this leads to so-called location and scale adjustments such as implemented in the dChip software (Li and Wong, 2003) under "using standardized separators".

© The Author 2017. Published by Oxford University Press. All rights reserved. For permissions, please e-mail: journals.permissions@oup.com

Johnson *and others* (2007) shrink the unwanted variation and variance of all genes within each batch using an empirical Bayes method. This leads to the widely used ComBat method which generally perform well in this case.

Walker *and others* (2008) propose a version of ComBat which uses replicate samples to estimate the batch effect. When replicate samples are available an alternative to centering, known as the ratio-based method (Luo *and others*, 2010) is to remove the average of the replicate samples within each batch rather than the average of all samples. Assuming that the factor of interest is associated with the batch, this should ensure that centering the batches does not remove the signal associated with the factor of interest.

1.2 *Observed factor of interest, unobserved unwanted factors*

Of course there is always a risk that some unknown sources also influence the gene expression. Furthermore it is sometimes better when tackling the problem with linear models to consider sources of unwanted variation which are actually known as unknown. The effect of these sources may be strongly non-linear because they don't affect all samples the same way or because they interact with other sources, in which case modeling them as known or simply additive may give poor results. When the sources of unwanted variation are modeled as unknown, the problem becomes more difficult because one has to estimate the unwanted factors along with their effects on the genes and because many estimates may explain the data equally well while leading to very different conclusions.

ICE (Intersample Correlation Emended, Kang *and others* (2008)) models unwanted variation as the combination of an observed fixed term and an unobserved random term. The covariance of the random effect is taken to be the covariance of the gene expression matrix. The risk of this approach is that some of the signal associated with the signal of interest may be lost because it is included in the covariance of the gene expression matrix.

SVA (Leek and Storey, 2007) addresses the problem by first estimating the effect of the factor of interest on each gene then doing factor analysis on the residuals, which gives good results as long as the unwanted factors are not too correlated with the factor of interest. Teschendorff *and others* (2011) propose a variant of SVA where the factor analysis step is done by independent component analysis (Hyvrinen and Oja, 2000) instead of singular value decomposition (SVD).

The same model as Leek and Storey (2007) is considered in a recent contribution of Gagnon-Bartsch and Speed (2012) coined RUV-2, which proposes a general framework to correct for unwanted variation in microarray data using negative *control genes*. These genes are assumed not to be affected by the factor of interest and are used to estimate the unwanted variation component of the model. Gagnon-Bartsch and Speed (2012) apply the method to several datasets in an extensive study and show its very good behavior for differential analysis, in particular comparable performances to state of the art methods such as ComBat (Johnson *and others*, 2007) or SVA (Leek and Storey, 2007).

Sun *and others* (2012) recently proposed LEAPP, which estimates the parameters of a similar model in two steps: first the effect of the unwanted factors is estimated by SVD on the data projected along the factor of interest, then the unwanted factors responsible for this effect and the effect of the factor of interest are estimated jointly using an iterative coordinate descent scheme. A sparsity-inducing penalty is added to the effect of the factor of interest in order to make the model identifiable.

Yang *and others* (2013) adopt a related approach: they also use the sparsity-inducing penalty, do not have the projection step and relax the rank constraint to a trace constraint which makes the problem jointly convex in the unwanted variation and effect of the factor of interest.

Listgarten *and others* (2010) model the unwanted variation as a random effect term, like ICE. The covariance of the random effect is estimated by iterating between a maximization of the likelihood of the factor of interest (fixed effect) term for a given estimate of the covariance and

a maximization of the likelihood of the covariance for a given estimate of the fixed effect term. This is also shown to yield better results than ICE and SVA.

1.3 *Unobserved factor of interest*

Finally when the factor of interest is not observed, the problem is even more difficult. It can occur if one is interested in unsupervised analyzes such as PCA or clustering. Suppose indeed that one wants to use a large study to identify new cancer subtypes. If the study contains several technical batches, includes different platforms or different labs or any unknown factor, the samples may cluster according to one of these sources hence defeating the purpose of using a large set of samples to identify more subtle subtypes. One may also simply want to "clean" a large dataset from its unwanted variation without knowing in advance which downstream analyses will be performed on the data. For example, in addition to clustering, survival or differential expression analyses may be carried out. Admittedly in the latter case, any knowledgeable person may want to start from the raw data and use the factor of interest once it becomes known to remove unwanted variation.

Alter *and others* (2000); Nielsen *and others* (2002) use SVD on gene expression to identify the unwanted factors without requiring the factor of interest, Price *and others* (2006) do so using axes of principal variance observed on SNP data. These approaches may work well in some cases but they rely on the prior belief that all unwanted factors explain more variance than any factor of interest. They will fail however if the unwanted factors are too correlated with the factor of interest. If the factor of interest is not observed but the unwanted factor is assumed to be an observed batch, an alternative approach is to project the data along the batch factors, equivalently to center the data by batch. This is conceptually similar to using one of the location and scale adjustment methods such as implemented in dChip Li and Wong (2003) or Johnson *and others* (2007) without specifying the factor of interest. Benito *and others* (2004);

Marron *and others* (2007) propose a distance weighted discrimination (DWD) method which uses a supervised learning algorithm to finds a hyperplane separating two batches and project the data on this hyperplane.

These approaches may lead to poor estimation of the variation of interest if it is correlated with the batch effect: if one of the batches contains most of one subtype and the second batch contains most of the other subtype the projection step removes a large part of the subtype signal. In addition, assuming that the unwanted variation is a linear function of the observed batch may fail if other unwanted factors affect gene expression or if the effect of the batch is a more complicated — possibly non-linear — function, or involves interaction with other sources.

Finally, Oncomine (Rhodes *and others*, 2004, 2007) regroups a large number of gene expression studies which are processed by median centering and normalizing the standard deviation to one for each array. This processing does not explicitly take into account a known unwanted factor or try to estimate it. It removes scaling effects, *e.g.* if one dataset or part of a dataset has larger values than others, but it does not correct for multivariate behavior such as the linear combination of some genes being larger for some batch : in particular if a single gene a has large values — compared to other genes — in a batch and low values in another batch, this will not be corrected. On the other hand, the correction does not run the risk of removing biological signal of this form.

2. Alternatives for the estimation of W

In the main paper, we consider the estimate $\hat{W}_2$ used in RUV-2, which relies on the SVD of the expression matrix restricted to its control genes. This estimate is shown to perform well for differential analysis tasks on an observed factor of interest (Gagnon-Bartsch and Speed, 2012). Unsupervised estimation of $X\beta$ may be more sensitive to the influence of the factor of interest X on the control genes: in the case of fixed α models, if the estimated $\hat{W}$ is very correlated with X in the sense of the canonical correlation analysis (Hotelling, 1936), *i.e.*, if there exists a linear

combination of the columns of X which has high correlation with a linear combination of the columns of $\hat{W}$, then most of the association of the genes with X will be lost by the correction. Random α models are expected to be less sensitive to the correlation of $\hat{W}$ with X but could be more sensitive to poor estimates of the variance carried by each direction of unwanted variation. This is also true regardless of the influence of X on the negative control genes if X is associated with the population W.

This suggests that unsupervised estimation methods could benefit from better estimates of W. We present here two directions that could lead to such estimators. The replicate-based correction introduced in Section 3 of the main manuscript yields yet another estimator of W.

2.1 *Using residuals*

In the case where X is observed, a common way of estimating W known as feasible generalized least squares (FGLS, Freedman (2005)) is to first do an ordinary regression of Y against X, then compute the empirical covariance on the residuals $Y - X\hat{\beta}$.

The estimators $\hat{W}_2$ of W that we introduced in Section 2 of the main manuscript works around the estimation of $X\beta$ by using genes for which β is known to be 0. Once we start estimating $X\beta$, *e.g.*, by iterating over $X\beta$ and α as described at the end of Section 2.3 of the main manuscript we can use a form of FGLS and re-estimate W using $Y - \hat{X\beta}$. If the current estimator of $X\beta$ is correct, this amounts to making all the genes control genes.

In practice, we use 100 iterations over $X\beta$ and α, and update W every 34 iterations, which amounts to performing three runs of about 30 ($X\beta$, α) iterations each, with updated W at each run. We found the procedure to be robust to changes in the iteration number, one simply needs to make sure that W is not updated in the last few iterations. This is what would happen if we did 100 iterations in total and updated after every 33 iterations: the last W update would occur at iteration 99, and the resulting α would not be optimized for the new W, which could lead to

poor estimation.

The total number of iterations is sometimes considered to be a regularization parameter itself in so called early stopping strategies (Prechelt, 1997) and can therefore be chosen using similar strategies as the ones we suggest for selecting the ridge parameter ν and the rank k of $W\alpha$ in Section 4. In practice, we found little difference when changing the number of iterations. Our `RUVnormalize` R package, the user is free to control the amount of optimization by specifying a maximum number of iterations, and a tolerance t such that optimization stops as soon as both $W\alpha$ and $X\beta$ changed by less than t after one iteration.

2.2 *Using a known W*

In some cases we may want to consider that W is observed. For example, if the dataset contains known technical batches, involves different platforms or labs, W could encode these factors instead of being estimated from the data. In particular if the corresponding W is a partition of the samples, then naively estimating α by regression using $X\beta = 0$ and removing $W\hat{\alpha}$ from Y corresponds to mean-centering the groups defined by W.

In most cases however, this procedure or its shrunken equivalent doesn't yield good estimates of $X\beta$. This was also observed by Gagnon-Bartsch and Speed (2012) for an observed factor of interest. One reason is that this W only accounts for known unwanted variation when other unobserved sources can influence the gene expression. The other one is that this approach leads to a linear correction for the unwanted variation in the representation used in W.

If we know that gene expression is affected by the temperature of the scanner, setting a column of W to be this temperature leads to a linear correction whereas the effect of the temperature may be quadratic, or involve interactions with other sources. In this case, estimating W implicitly allows us to do a non-linear correction because the estimated W could fit any non-linear representation of the observed unwanted variation which actually affects gene expression.

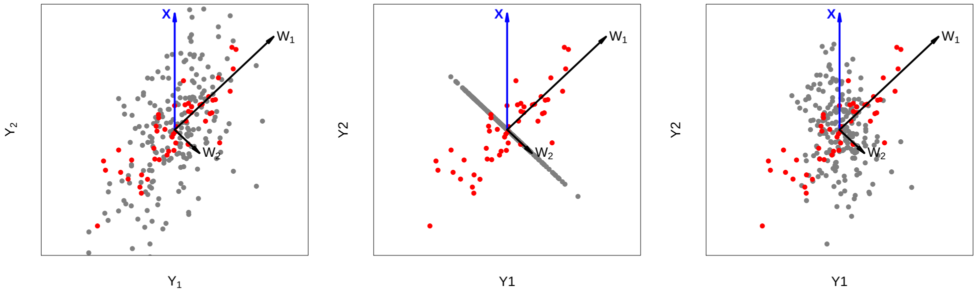

Fig. 1. Naive RUV-2 (fixed α) and random α based corrections, with $m = 2$.

3. Naive fixed α RUV-2 vs random α RUV-2

As discussed in Section 2 of the main manuscript, the only difference between the naive RUV-2 estimator of Gagnon-Bartsch and Speed (2012) and the newly introduced random α RUV-2 is the ℓ_2 penalty term: the former is a ridge regression against $\hat{W}_2$ — maximum a posteriori for a random α model– whereas the latter is an ordinary regression – maximum likelihood for a fixed α model. In this context where X is unobserved and $X\beta$ is set to 0 to estimate α, this difference can be important if X and W are correlated.

Figure 1 shows an example of such a case. The left panel represents genes for $m = 2$. Red dots are control genes, gray ones are regular genes. The largest unwanted variation W_1 correlates with the factor of interest X.

In naive RUV-2 with $k = 1$, the correction projects the samples in the orthogonal space of W_1, which can remove a lot of the signal coming from the factor of interest. This is illustrated on the center panel which shows the data corrected by naive RUV-2, *i.e.*, by projecting the genes on the orthogonal space of W_1. The projection removes all effect coming from W_1 but also greatly reduces the association of genes with X. This is true regardless of the amount of variance actually caused by W_1: the result would be the same with an almost spherical unwanted variation $\|W_1\| \simeq \|W_2\|$ because once W is identified, the projection step of naive RUV-2 does not take

into account any variance information. On the other hand, the projection does not account at all for the unwanted variation along W_2.

By contrast, the random α correction shown on the right panel of Figure 1 takes variance into account. The ridge regression removes only a limited amount of signal along each unwanted variation direction, proportional to the amount of variance that was observed in the control genes.

4. Choice of the ridge parameter ν on gender data

As mentioned in Section 5.1 of the main manuscript, choosing the ridge parameter ν is a difficult problem in general. In supervised tasks like classification, regularization parameters are often chosen using cross-validation or hold-out procedures. For normalization or clustering tasks on the other hand these procedures cannot be used, and different values of ν will lead to different corrected datasets, which are hard to compare. In this Section, we show two indicators which can be used to choose ν in general: RLE plots, and positive control genes. In the context of this specific experiment (Section 5.2 of the main manuscript), the only honest way to pick ν would be by using RLE plots, but we still discuss others like positive control genes and clustering error as they can be useful for other applications.

4.1 *RLE plots*

RLE plots represent each array by a boxplot of the log intensities of its probes. Each log intensity corrected by the median log intensity of the probe across all arrays. The fact that arrays have very different boxplots can indicate the presence of unwanted variation. In addition, if all IQRs become small, *i.e.*, most probes are very close to their median, we can suspect that too much signal has been removed by the normalization procedure. Unpublished experiments in our group suggest that clean replicates of the same sample lead to RLE plots with IQR around 0.1 or 0.05. Datasets formed by different biological samples are expected to have larger IQRs. However this

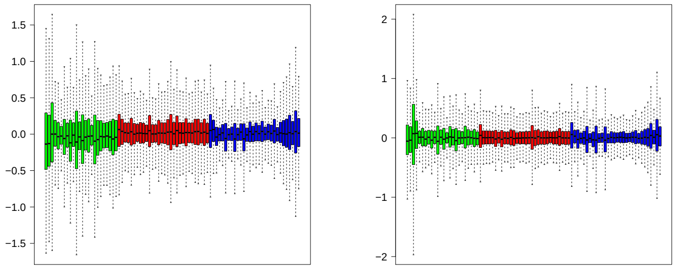

(a) Uncorrected (b) Centered

Fig. 2. RLE plots for the gender data, uncorrected and after mean centering. Colors correspond to labs: green is UC Davis , red is UC Irvine and blue is University of Michigan, Ann Arbor.

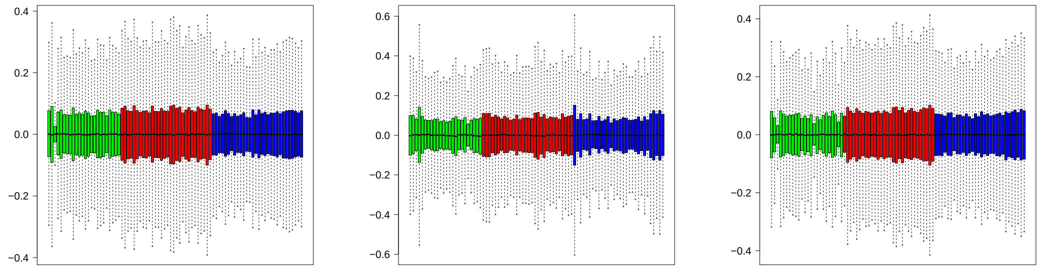

(a) Naive RUV-2 (b) Replicate based (c) Replicate based with iterations

Fig. 3. RLE plots for the gender data after naive RUV-2, replicate based and replicate + iterations corrections. Colors correspond to labs: green is UC Davis , red is UC Irvine and blue is University of Michigan, Ann Arbor.

approach can be misleading if the variation of interests involves only a few genes: if most genes do not vary, IQRs can be very small.

Figures 2 and 3 show RLE plots before correction, after mean centering, naive RUV-2, and replicate based corrections. The uncorrected data contains strong unwanted variation, leading to boxes with very differents medians and amplitudes. All corrections lead to IQRs larger than 0.1. Figure 4 and 5 show RLE plots after correction with random α without and with iterations respectively, and for various values of ν. As expected, smaller values of ν lead to smaller IQRs, as

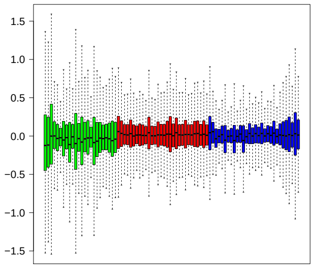

(a) $\nu = 10.\sigma_1(WW^\top)$

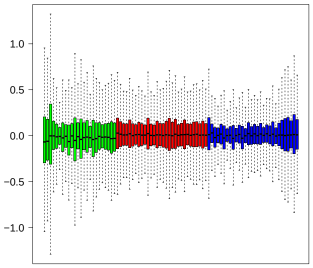

(b) $\nu = \sigma_1(WW^\top)$

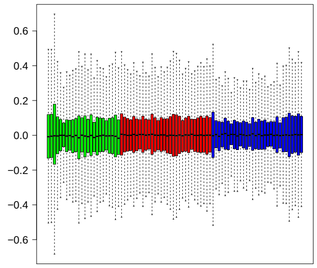

(c) $\nu = 10^{-1}\sigma_1(WW^\top)$

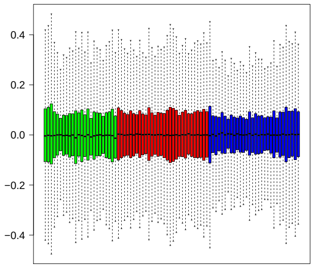

(d) $\nu = 5.10^{-2}\sigma_1(WW^\top)$

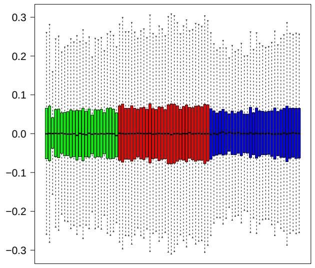

(e) $\nu = 10^{-2}\sigma_1(WW^\top)$

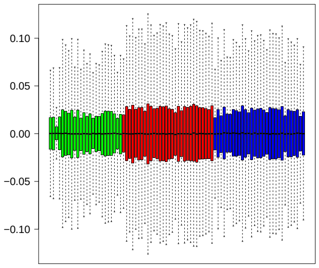

(f) $\nu = 10^{-3}\sigma_1(WW^\top)$

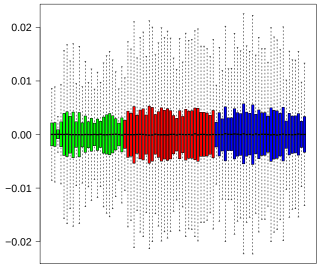

(g) $\nu = 10^{-4}\sigma_1(WW^\top)$

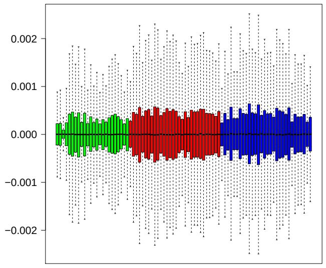

(h) $\nu = 10^{-5}\sigma_1(WW^\top)$

Fig. 4. RLE plots for the gender data after naive random RUV correction with different shrinkage levels. Colors correspond to labs: green is UC Davis , red is UC Irvine and blue is University of Michigan, Ann Arbor.

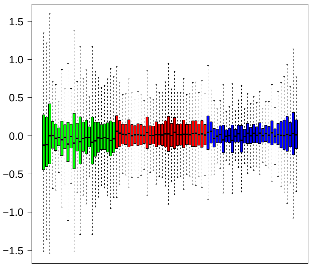

(a) $\nu = 10.\sigma_1(WW^\top)$

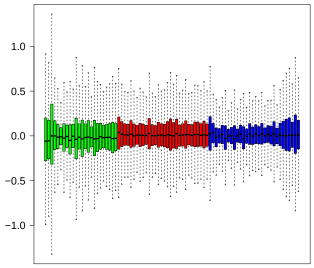

(b) $\nu = \sigma_1(WW^\top)$

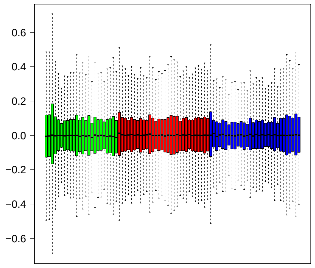

(c) $\nu = 10^{-1}\sigma_1(WW^\top)$

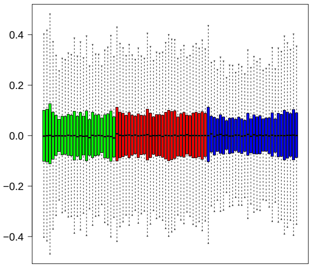

(d) $\nu = 5.10^{-2}\sigma_1(WW^\top)$

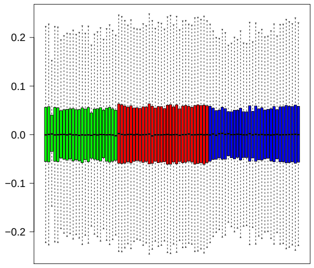

(e) $\nu = 10^{-2}\sigma_1(WW^\top)$

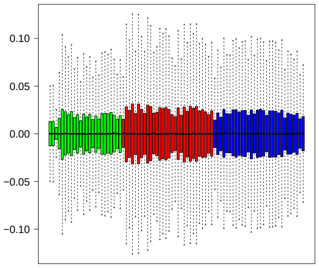

(f) $\nu = 10^{-3}\sigma_1(WW^\top)$

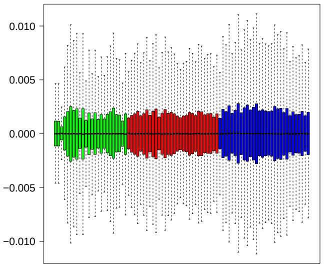

(g) $\nu = 10^{-4}\sigma_1(WW^\top)$

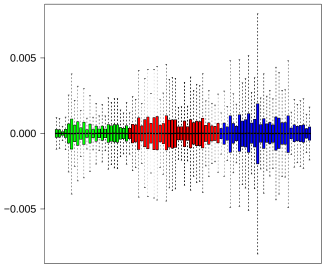

(h) $\nu = 10^{-5}\sigma_1(WW^\top)$

Fig. 5. RLE plots for the gender data corrected by iterated random RUV with different shrinkage levels. Colors correspond to labs: green is UC Davis , red is UC Irvine and blue is University of Michigan, Ann Arbor.

more variance is removed. Both with and without iterations, $\nu = 10^{-2}\sigma_1(WW^\top)$ is the smallest value leading to IQRs larger than 0.1.

4.2 *Positive control genes and clustering error*

	Clustering error	20	40	60
Uncorrected	1.000	13	13	14
Centered	0.973	12	18	18
Naive RUV-2	0.751	14	19	24
Naive + shrink	0.427	16	22	27
Replicates	0.770	16	23	29
Replicates + iter	0.486	19	25	28
Shrinkage + iter	0.091	15	22	26

Table 1. Clustering errors and genes on the X and Y chromosomes among the first 20, 40 and 60 DE genes as a function of the number of selected genes for various correction methods.

ν	Clustering error	20	40	60
$10.\sigma_1(WW^\top)$	1.000	13	13	14
$\sigma_1(WW^\top)$	0.998	14	17	17
$10^{-1}\sigma_1(WW^\top)$	0.998	15	21	24
$5.10^{-2}\sigma_1(WW^\top)$	0.851	16	22	26
$10^{-2}\sigma_1(WW^\top)$	0.427	16	22	27
$10^{-3}\sigma_1(WW^\top)$	0.674	15	19	20
$10^{-4}\sigma_1(WW^\top)$	0.932	10	11	11
$10^{-5}\sigma_1(WW^\top)$	0.955	7	8	8

Table 2. Clustering errors and genes on the X and Y chromosomes among the first 20, 40 and 60 DE genes as a function of the number of selected genes for naive RUV-2 corrected data with different shrinkage levels.

Another possible way to assess which ν leads to better normalizations is to count how many genes known to be differentially expressed for a known factor of interest are indeed detected as such after each normalization. This approach was used in Gagnon-Bartsch and Speed (2012). It implies by definition that we know a biological signal of interest, and a few differentially expressed genes. This may not be the case for all datasets, and even when this type of information is available it is only a guideline since detecting these positive control genes does not guarantee that other signals of interest are maintained.

ν	Clustering error	20	40	60
$10.\sigma_1(WW^\top)$	1.000	13	13	13
$\sigma_1(WW^\top)$	0.998	14	16	17
$10^{-1}\sigma_1(WW^\top)$	0.998	16	20	22
$5.10^{-2}\sigma_1(WW^\top)$	0.761	15	21	22
$10^{-2}\sigma_1(WW^\top)$	0.091	15	22	26
$10^{-3}\sigma_1(WW^\top)$	0.179	14	18	20
$10^{-4}\sigma_1(WW^\top)$	0.305	7	8	8
$10^{-5}\sigma_1(WW^\top)$	0.903	3	3	3

Table 3. Clustering errors and genes on the X and Y chromosomes among the first 20, 40 and 60 DE genes as a function of the number of selected genes for RUV-2+iteration corrected data with different shrinkage levels.

Table 2 and 3 show the number of detected positive control genes for increasing values of ν, without and with iterations respectively. The factor of interest considered is gender and the positive control genes are the ones located on chromosomes X and Y — Table 1 shows the same thing for other methods, for comparison purpose. In both cases, the $\nu = 10^{-2}\sigma_1(WW^\top)$ value suggested by the analysis of RLE plots leads to the largest number of detected positive control genes. The tables also show the clustering error (after keeping the 1260 largest variance genes) for each normalization. For this criterion again $\nu = 10^{-2}\sigma_1(WW^\top)$ leads to the best correction possible among the assessed values.

Admittedly, measuring how well the data cluster by gender — like we do in Section 5.2 of the main manuscript — after using genes on the X and Y chromosomes — or worse, using the clustering error itself — to choose ν would be over optimistic. The goal here is rather to discuss and illustrate different ways to assess how well a normalization performs. In our case, all criteria, including RLE which does not require the knowledge of a known factor of interest, agree on what the best normalization is.

4.3 *PCA plots for different shrinkage levels*

In Section 5.2, we measure how each normalization method performs by assessing how well the data cluster by gender after normalization — the clustering error displayed on Table 2 and 3 — and by visual inspection of PCA plots. Figures 6 and 7 show these PCA plots for naive and iterated random RUV respectively, with increasing levels of shrinkage. Consistently with Table 2 and 3, we observe that $\nu = 10^{-2}\sigma_1(WW^\top)$ leads to a better clustering by gender than $\nu = 5.10^{-2}\sigma_1(WW^\top)$, and that normalization using less shrinkage starts removing gender effect as well.

Finally, Figures 8 and 9 show the brain region factor on PCA plots for naive and iterated random RUV respectively, with increasing levels of shrinkage. It is visually very clear that for larger values of ν (between $5.10^{-2}\sigma^2(WW^\top)$ and $\sigma^2(WW^\top)$), the main effect in the data is brain region, more specifically cerebellar hemisphere samples ('c', in green) versus anterior cingulate ('a', in red) and dorsolateral prefrontal cortex ('d', in blue) ones. For $\nu = 10.\sigma_1(WW^\top)$, this effect is still present along the second principal component, but the main effect is the lab: in this case, the data is practically uncorrected, as seen by comparison with the left panel of Figure 4 in the main manuscript. This illustrates a crucial point: looking for the right value of ν — or more generally of amount of variance to be removed — does not make sense in general. In this case, smaller values of ν are good to recover the gender signal of the dataset, either by clustering or differential expression analysis. If, on the other hand, one is interested by the brain region signal, larger values of ν are preferable. In practice on new data, it is therefore important to try different values of ν and analyse the results obtained after each adjustment.

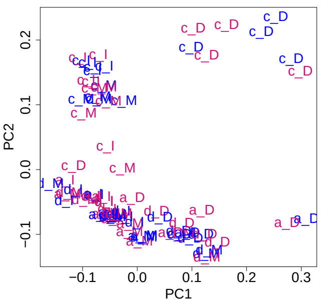

(a) $\nu = 10.\sigma_1(WW^\top)$

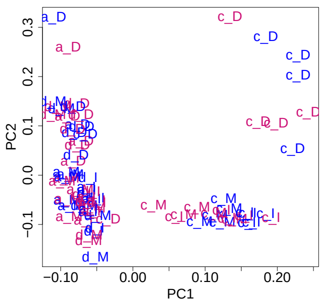

(b) $\nu = \sigma_1(WW^\top)$

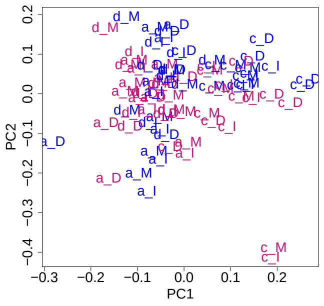

(c) $\nu = 10^{-1}\sigma_1(WW^\top)$

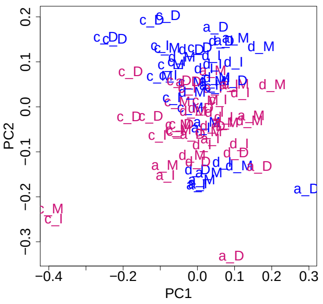

(d) $\nu = 5.10^{-2}\sigma_1(WW^\top)$

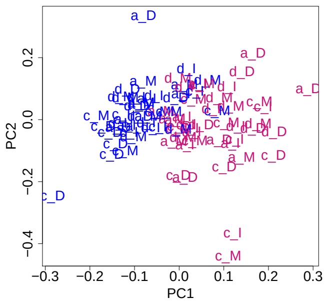

(e) $\nu = 10^{-2}\sigma_1(WW^\top)$

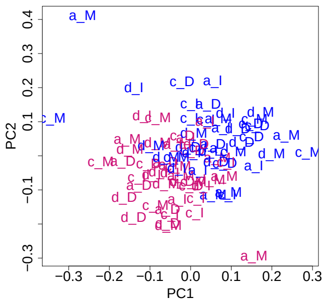

(f) $\nu = 10^{-3}\sigma_1(WW^\top)$

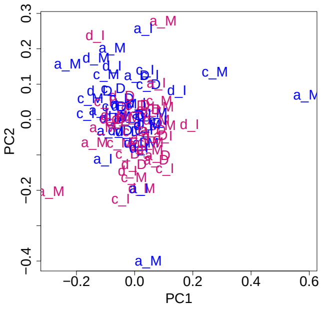

(g) $\nu = 10^{-4}\sigma_1(WW^\top)$

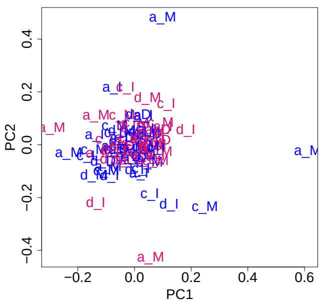

(h) $\nu = 10^{-5}\sigma_1(WW^\top)$

Fig. 6. PCA plots of the gender data after a naive random RUV correction with different shrinkage levels. Colors represent gender: pink for female, blue for male. Minuscule letters are brain regions: anterior cingulate cortex (a), dorsolateral prefontal cortex (d) and cerebellar hemisphere (c). Capital letters are labs: UC Irvine (I), UC Davis (D) and University of Michigan, Ann Arbor (M).

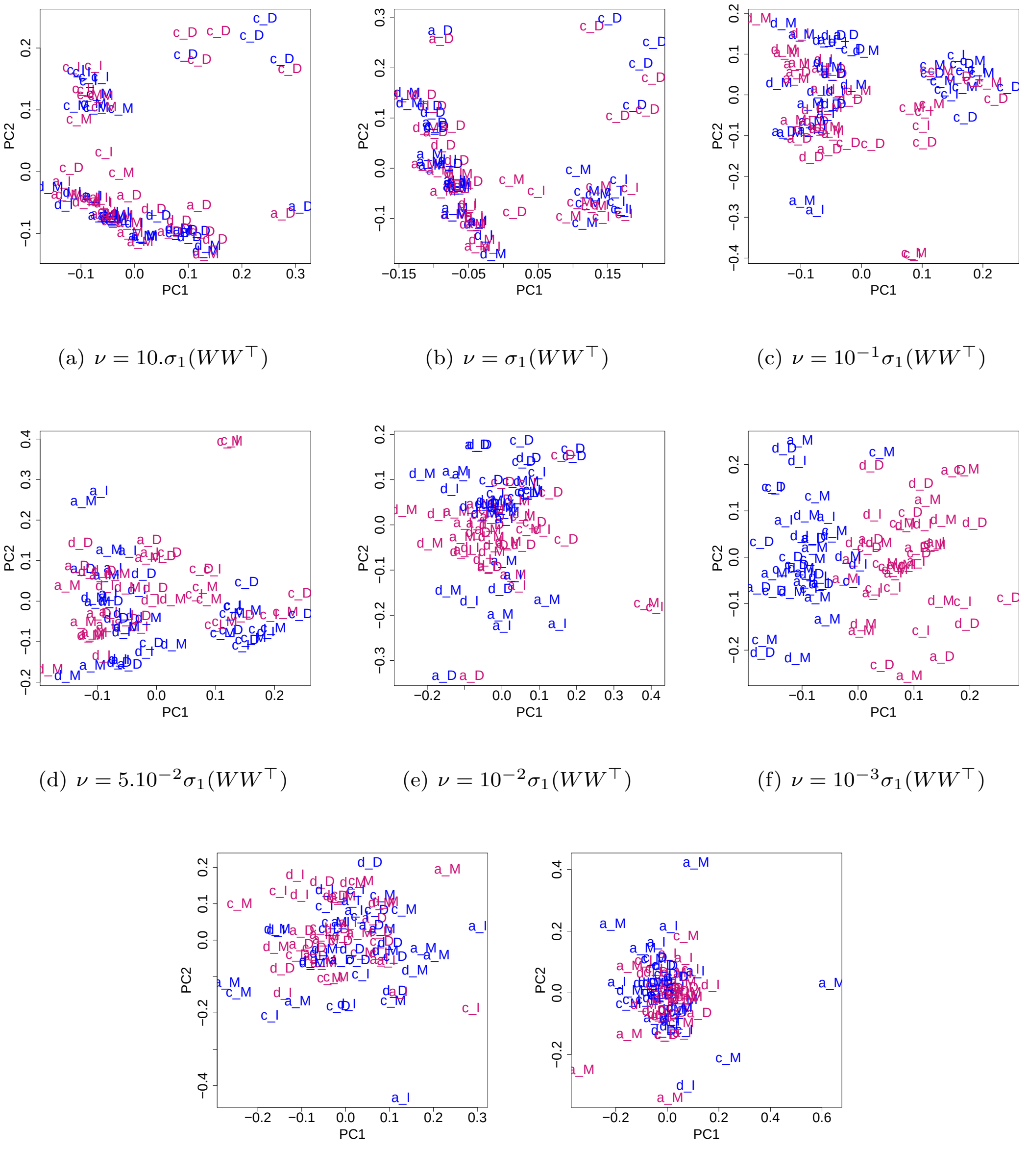

(a) $\nu = 10.\sigma_1(WW^\top)$ (b) $\nu = \sigma_1(WW^\top)$ (c) $\nu = 10^{-1}\sigma_1(WW^\top)$

(d) $\nu = 5.10^{-2}\sigma_1(WW^\top)$ (e) $\nu = 10^{-2}\sigma_1(WW^\top)$ (f) $\nu = 10^{-3}\sigma_1(WW^\top)$

(g) $\nu = 10^{-4}\sigma_1(WW^\top)$ (h) $\nu = 10^{-5}\sigma_1(WW^\top)$

Fig. 7. PCA plots of the gender data, after iterated random RUV correction with different shrinkage levels. Colors represent gender: pink for female, blue for male. Minuscule letters are brain regions: anterior cingulate cortex (a), dorsolateral prefontal cortex (d) and cerebellar hemisphere (c). Capital letters are labs: UC Irvine (I), UC Davis (D) and University of Michigan, Ann Arbor (M).

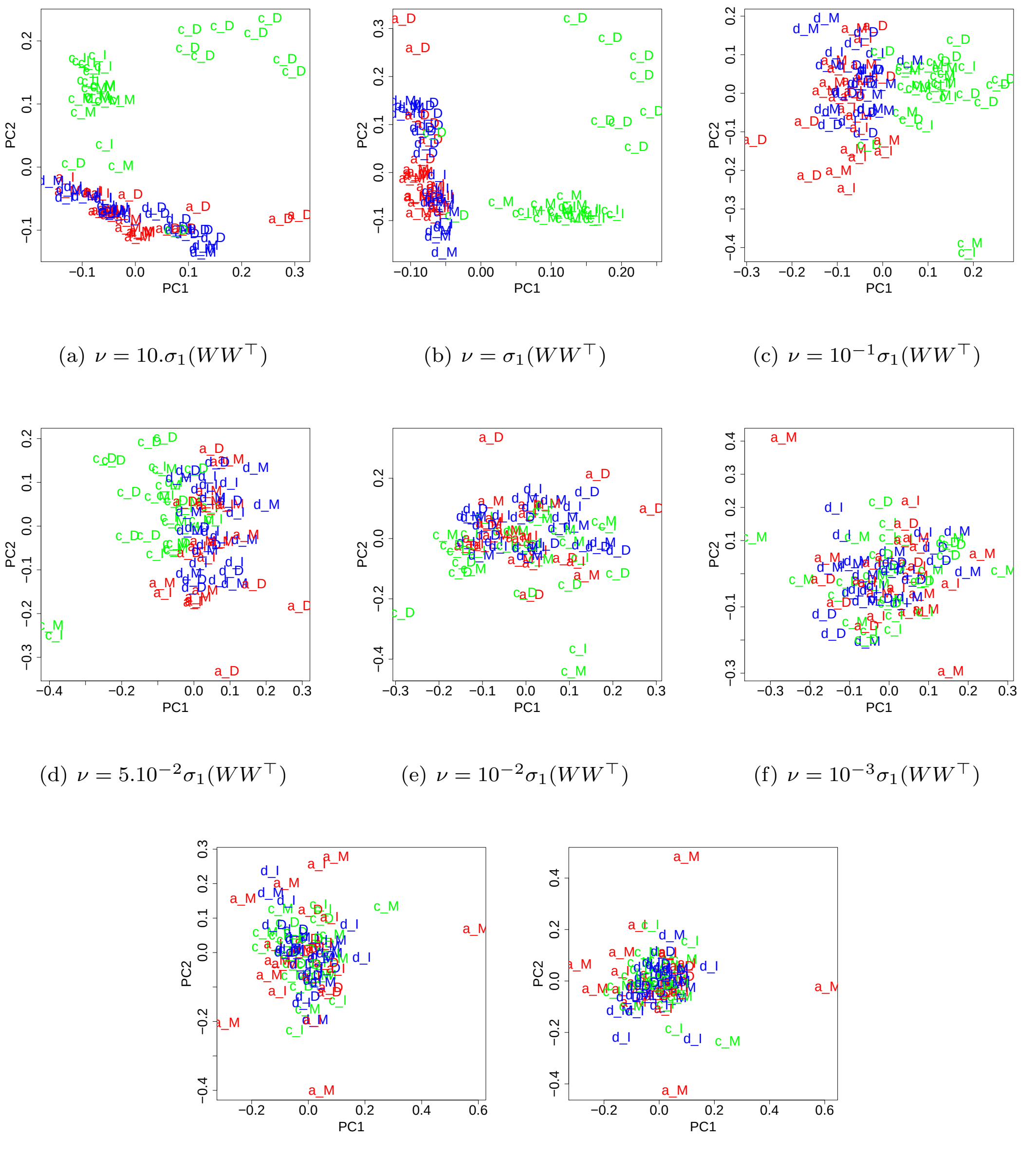

(a) $\nu = 10.\sigma_1(WW^\top)$ (b) $\nu = \sigma_1(WW^\top)$ (c) $\nu = 10^{-1}\sigma_1(WW^\top)$

(d) $\nu = 5.10^{-2}\sigma_1(WW^\top)$ (e) $\nu = 10^{-2}\sigma_1(WW^\top)$ (f) $\nu = 10^{-3}\sigma_1(WW^\top)$

(g) $\nu = 10^{-4}\sigma_1(WW^\top)$ (h) $\nu = 10^{-5}\sigma_1(WW^\top)$

Fig. 8. PCA plots of the gender data, after naive random RUV correction with different shrinkage levels. Colors and minuscule letters represent brain regions: anterior cingulate cortex (red, a), dorsolateral prefontal cortex (blue, d) and cerebellar hemisphere (green, c). Capital letters are labs: UC Irvine (I), UC Davis (D) and University of Michigan, Ann Arbor (M).

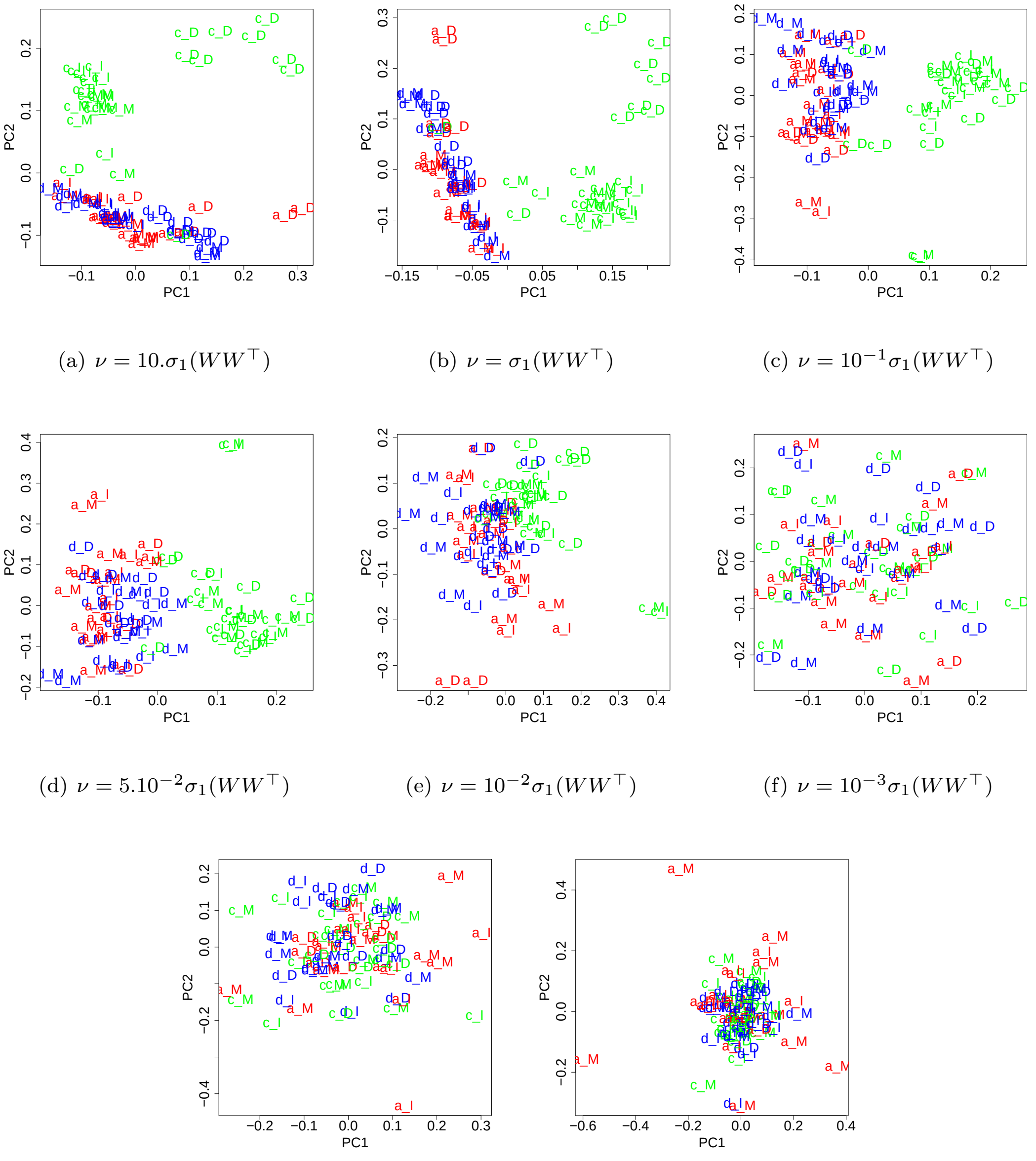

(a) $\nu = 10.\sigma_1(WW^\top)$ (b) $\nu = \sigma_1(WW^\top)$ (c) $\nu = 10^{-1}\sigma_1(WW^\top)$

(d) $\nu = 5.10^{-2}\sigma_1(WW^\top)$ (e) $\nu = 10^{-2}\sigma_1(WW^\top)$ (f) $\nu = 10^{-3}\sigma_1(WW^\top)$

(g) $\nu = 10^{-4}\sigma_1(WW^\top)$ (h) $\nu = 10^{-5}\sigma_1(WW^\top)$

Fig. 9. PCA plots of the gender data, after iterated random RUV correction with different shrinkage levels. Colors and minuscule letters represent brain regions: anterior cingulate cortex (red, a), dorsolateral prefontal cortex (blue, d) and cerebellar hemisphere (green, c). Capital letters are labs: UC Irvine (I), UC Davis (D) and University of Michigan, Ann Arbor (M).

5. TCGA glioblastoma data

5.1 *Data*

We now illustrate the performances of our method on the gene expression array data generated in the TCGA project for glioblastoma (GBM) tumors (Cancer Genome Atlas Research Network, 2008). These tumors were studied in detail in Verhaak *and others* (2010). For each of the 460 samples, gene expression was measured on three different platforms: Affymetrix HT-HG-U133A Genechips at the Broad Institute, Affymetrix Human Exon 1.0 ST Genechips at Lawrence Berkeley Laboratory and Agilent 244K arrays at University of North Carolina. Verhaak *and others* (2010) selected 200 tumors and 2 normal samples from the dataset based on sample quality criterions and filtered 1740 genes based on their coherence among the three platforms and their variability within each platform.

The expression values from the three platforms were then merged using factor analysis. They identified four GBM subtypes by clustering analysis on this restricted dataset: Classical, Mesenchymal, Proneural and Neural. We study these 202 samples across the three platforms, keeping all the 11861 genes in common across the three platforms. Among these 202 samples, 173 were identified by Verhaak *and others* (2010) as “core” samples: they were good representers of each subtypes. 38 of them are Classical, 56 Mesenchymal, 53 Proneural and 26 Neural.

5.2 *Design*

For the purpose of the experiment, we study how well a particular correction allows us to recover the correct label of the 147 Classical, Mesenchymal and Proneural tumors, leaving the other ones aside. Our objective is to recover the correct subtypes using a k-means with 3 clusters.

We consider two settings. In the first one, we use a full design with all 147 samples from 3 platforms. In the second one we build a confounding setting in which we only keep the Classical

samples on Affymetrix HT-HG-U133A arrays, the Mesenchymal samples on Affymetrix Human Exon arrays and the Proneural samples on Agilent 244K arrays. In each case, we use 5 randomly selected samples that we keep for all 3 platforms and use as replicates.

We do not use other samples as replicates even in the full design when all samples could potentially be used as replicates. Among the 5 selected samples one was Neural, two Proneural, and two were not assigned a subtype. The results presented are qualitatively robust to the choice of these replicates.

In the confounded design, a correction which simply removes the platform effect is likely to also lose all the subtype signal because it is completely confounded with the platform, up to the replicate samples. The reason why we only keep 3 subtypes is to allow such a total confounding of the subtypes with the 3 platforms. In this design however, applying no correction at all is likely to yield a good clustering by subtype because we expect the platform signal to be very strong.

A good correction method should therefore perform well in both the confounded and the full design. In the full design, the platform effect is orthogonal to the subtype effect so we expect the correction to be easier. Of course in this case, the uncorrected data is expected to cluster by platform which this time is very different from the clustering by subtype since each sample is present on each platform.

5.3 *Result*

Table 4 shows the clustering error obtained for each correction method on the two designs. Recall that since there are 3 clusters, clustering errors range between 0 and 2. As expected, the uncorrected data give a maximal error on the full design and 0 in the presence of confounding. This is because, as seen on Figure 10, the uncorrected data cluster by platform which in the full design are orthogonal to the subtypes and in the second design are confounded with subtypes.

For similar reasons, centering the data by platform works well in the full design but fails

Method	Full	Confounding
No correction	2	0
Mean-centering	0.3	1.93
Ratio method	0.31	0.79
Naive RUV-2	2	0
Random α	0.21	1.5
+ iterations	0.15	1
Replicate based	0.2	0.61
+ iterations	0.17	0.16

Table 4. Clustering error of TCGA GBM data with full and confounded designs for various correction methods. Since there are 3 clusters, errors range between 0 and 2.

when there is confounding because removing the platform effect removes most of the subtype effect. When replicates are available, a variant of mean centering is to remove the average of replicate samples from each platform. This is known as the ratio method (Luo *and others*, 2010) and does improve on regular mean-centering in the presence of confounding. A disadvantage of this method is that it amounts to considering that W is a partition of the data by batch (in this case by platform) whereas as discussed in Section 2 of this supplementary material, the actual unwanted variation may be a non linear function of the batch, possibly involving other sources. Note that we do not explicitly assess the ratio method for the other benchmarks because all samples are used as replicates so removing the average of replicate samples from each batch amount to centering the samples by batch and the ratio method becomes equivalent to mean centering.

Naive RUV-2 gives a maximal error in the full design, 0 otherwise. This result is actually caused by the fact that naive RUV-2 is extremely sensitive to the choice of k. Since the total number of differences formed on the 15 replicates is 10, we use $k = 2$ as a default for naive RUV-2 and the replicate based procedure. In the full design, the platform effect is contained in the first three principal components of the control gene matrix Y_c and the fourth principal component contains the subtype effect. This can clearly be seen on Figure 10. Removing only one or two directions of variance leaves too much platform effect. Removing a third one ($k = 3$) gives a small

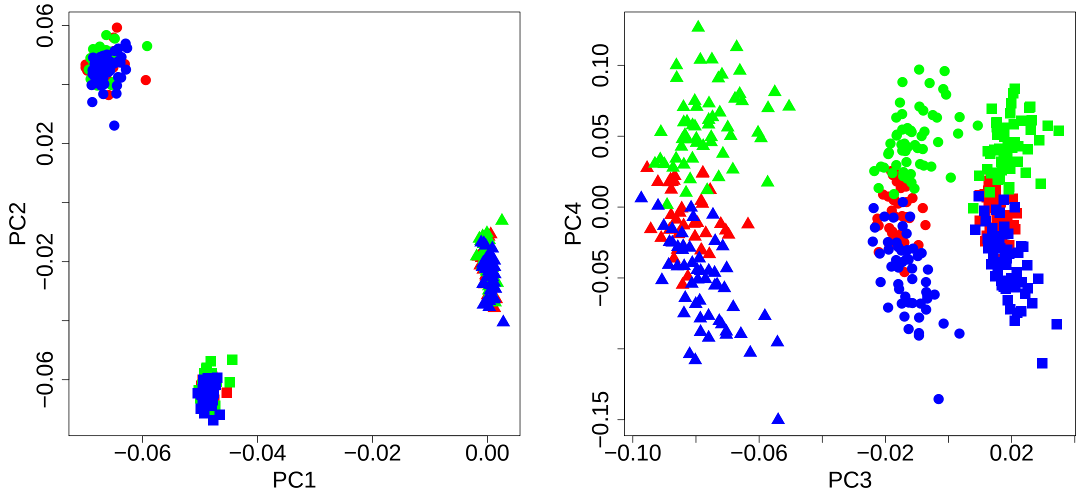

Fig. 10. Uncorrected full design GBM data in the space of their first four principal components. Left panel: PC 1 and 2, right panel: PC 3 and 4. Colors denote subtypes, shapes denote platforms.

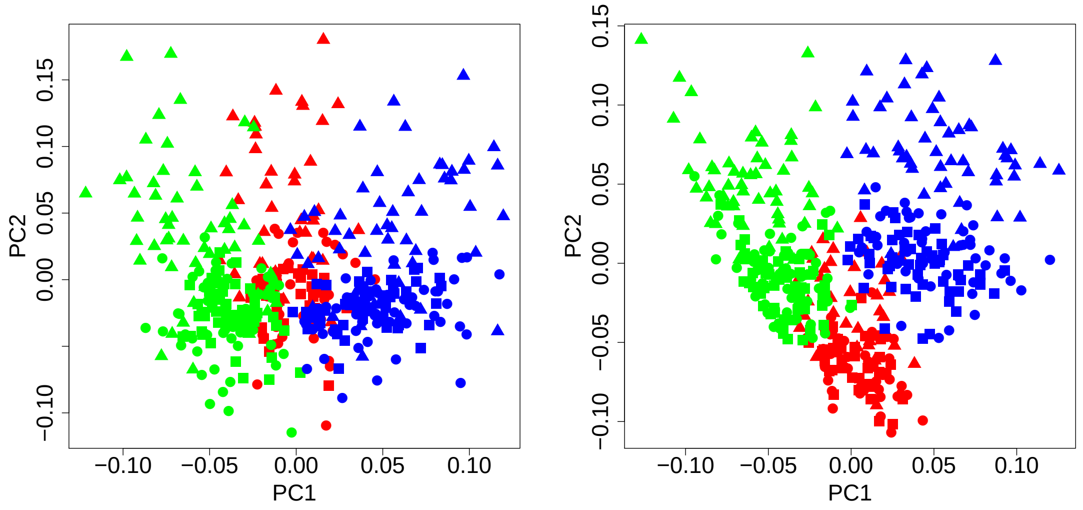

Fig. 11. GBM full design random α with (left) and without (right) iterations. Colors represent subtypes, shapes represent platforms.

error of 0.15 and removing a fourth one gives an error of 1.83.

When the platform is confounded with the subtype, removing one or two components leads to a perfect clustering by subtype because the third principal component still contains plat-

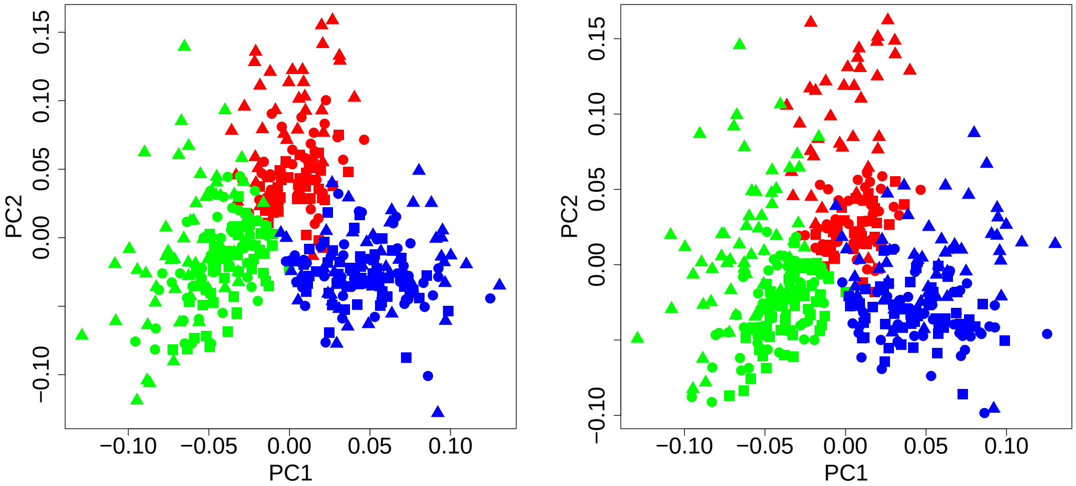

Fig. 12. GBM full design replicate-based with (left) and without (right) iterations. Colors represent subtypes, shapes represent platforms.

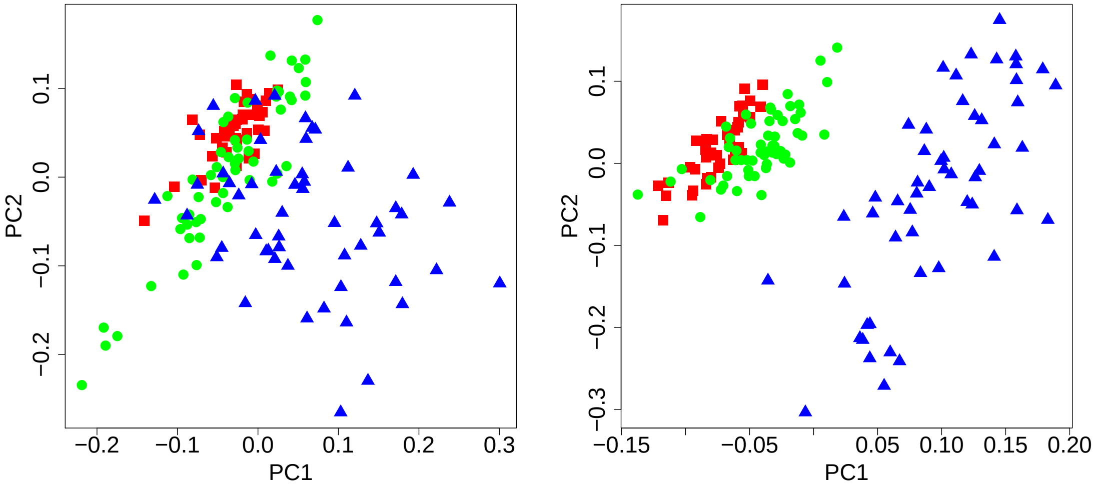

Fig. 13. GBM confounded design random α with (left) and without (right) iterations. Colors represent subtypes, shapes represent platforms.

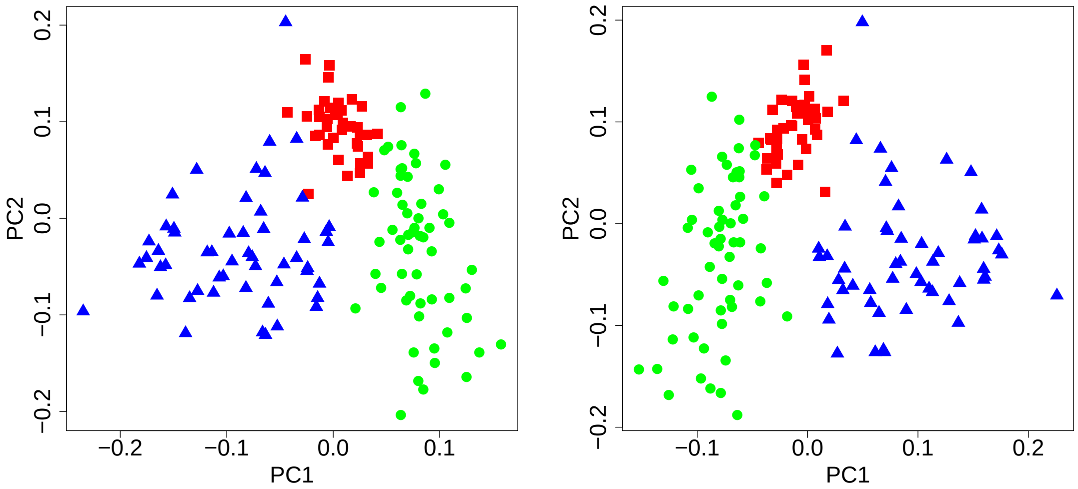

Fig. 14. GBM confounded design replicate-based with (left) and without (right) iterations. Colors represent subtypes, shapes represent platforms.

form/subtype signal. Removing more does not allow us to recover a clustering by subtype. So if we used $k = 3$ instead of $k = 2$, the result would be inverted: good for the full design, bad in the presence of confounding.

The random α model works well in the full design, less so in the presence of confounding, as illustrated on Figure 11 and 13 respectively. While it was reasonably robust to the choice of the ridge parameter ν on the gender data, it is more sensitive on this one. Using $\nu = \sigma_1(W^\top W) \times 5.10^{-2}$ instead of 10^{-3} does not remove enough platform effect and leads to an error of 2 on the full design, 0 in the presence of confounding. Using a smaller factor of 5.10^{-4} leads to an error of 0.27 (1.65 with confounding), and 10^{-4} to an error of 1.94 (1.98 with confounding).

Because the correction made by the random α model is softer than the one of the fixed α naive RUV-2, using $\nu = \sigma_1(W^\top W) \times 10^{-3}$ allows us to recover subtype signal in both designs. The sensitivity to ν is likely to be caused by the large difference of magnitude between $\sigma_1(W^\top W)$ and the next eigen values: the first one represents 98% of the total variance. This is to be expected in most cases in presence of a strong technical batch effect.

In both designs, iterating between estimation of $X\beta$ using sparse dictionary learning and estimation of α using ridge regression further improves the performances. The replicate-based correction gives good results for both designs, as illustrated on Figure 12 and 14. Like for the gender data, it seems to be robust to the choice of k. For $k = 10$ it gives errors 0.2 and 0.53 in the first and second design respectively. Here again, adding iterations on $(X\beta, \alpha)$ improves the quality of the correction in each case.

As with the gender data, the difference observed between the correction methods cannot be explained solely by the fact that some of them remove more variance than others. For example in the full design, naive RUV-2, the replicate based procedure and the random α correction lead to similar $\|W\alpha\|_F$ but to very different performances: naive RUV-2 fails to remove enough platform signal whereas the other corrections remove enough of it for the arrays to cluster by subtype.

In the confounding design, both naive RUV-2 and the replicate based procedure lead to similar $\|W\alpha\|_F$ and similar performances. The random α correction leads to a larger $\|W\alpha\|_F$ which explains its poor behavior. Estimating what amount of variance should be removed is part of the problem, so it would not be correct to conclude that the random α correction works as well as the others in this case. It is however interesting to check whether the problem of a particular method is its removing too much or not enough variance or whether it is a qualitative problem.

6. MAQC-II data

We finally assess our correction methods on a gene expression dataset which was generated in the context of the MAQC-II project (Shi *and others*, 2010). The study was done on rats and the objective was to assess hepatotoxicity of 8 drugs. For each drug, three time points were done (animals were sacrificed 6, 24 or 48 hours after drug injection) for three different doses : low, medium and high. For each of these $8 \times 3 \times 3$ combinations, 4 animals were tested for a total of 288 animals. For each animal, one blood and one liver sample were taken. Gene expression in

blood and in the liver were measured using Agilent arrays and gene expression in the liver was also measured using Affymetrix arrays.

The Agilent arrays were loaded using the `marray` R package. Each array was loess normalized, dye swaps were averaged and each gene was then assigned the median log ratio of all probesets corresponding to the gene. The Affymetrix arrays were normalized using the `gcrma` R package. Each gene was then assigned the median log ratio of all probesets corresponding to the gene. For this experiment we retain samples from all platforms and tissues for the highest dose of each drug and for the last two time points 24 hours and 48 hours. Most of these drugs are not supposed to be effective for the earlier time points. This leads to a set of 186 arrays that we restrict to the 9502 genes which are common to all platforms. Each sample has a replicate for each tissue and platform, but there is no replicate against the time effect. For control genes, we used the same list of housekeeping genes as for the other datasets but converted to their rat orthologs, leading to 210 control genes.

The interest of this complex design is obvious for the purpose of this paper: the resulting dataset contains a large number of arrays measuring gene expression influenced by the administered drug which we consider to be our factor of interest and by numerous sources of unwanted factors. Array type, tissue, time and dose are likely to influence gene expression, preventing the arrays from clustering by drug.

This clustering problem is much harder than the gender and glioblastoma ones. First of all, the drug signal may not be as strong as the gender which at least for a few genes is expected to be very clear or as the glioblastoma subtypes which were defined on the same dataset. Second and maybe more important, it is an 8-class clustering problem, which is intrinsically harder than 2- or 3-class clusterings. Finally as we discuss in Section 7 of this supplementary material, the control genes for this dataset do not behave as expected.

The errors obtained after applying each correction are displayed in Table 5. Recall that for this

Method	Error
No correction	5.9
Mean-centering	5.1
Naive RUV-2	6.6
Random α	4.7
+ iteration	5.4
Replicate based	2.8 – 3.8
+ iterations	2.8 – 3.8

Table 5. Clustering error of MAQC-II data for various correction methods.

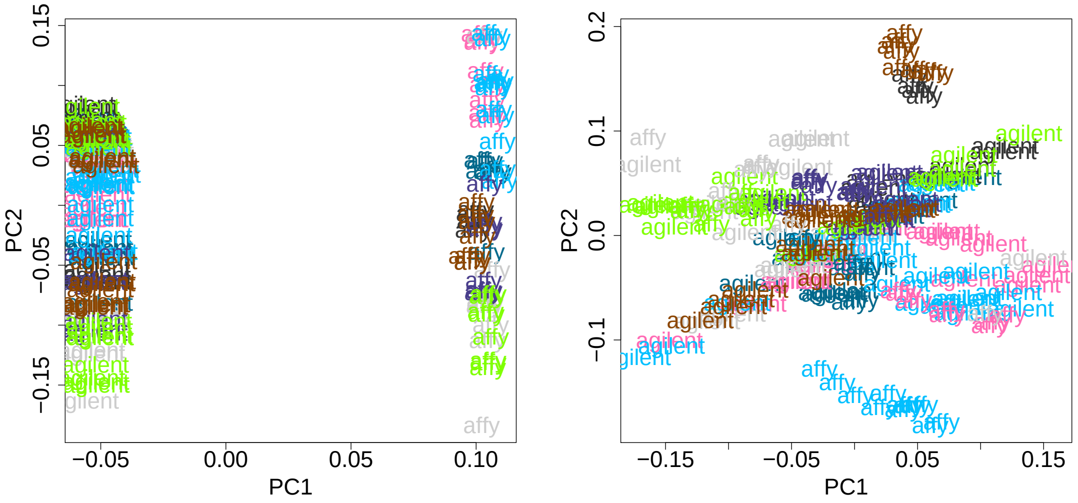

Fig. 15. Samples of the MAQC-II study represented in the space of their first two principal components before correction (left panel) and after centering by tissue and platform region (right panel). Each color represents a different drug. The labels indicate the platform of each sample.

dataset, we are trying to recover a partition in 8 classes corresponding to the 8 drugs of the study so the maximum clustering error is 7. The left panel of Figure 15 represents the uncorrected samples in the space of the first two principal components. The first principal components is clearly driven by the presence of two different types of arrays. The clustering error in this case is 5.9.

Centering by platform-tissue, *i.e.* centering separately the Affymetrix arrays, the Agilent liver and the Agilent blood, the data points do not cluster by platform anymore but just like for the gender data this does not lead to a clear clustering by drug. This can be seen on the right panel

of Figure 15. The resulting clustering error is 5.1. The naive RUV-2 correction doesn't lead to any improvement compared to the uncorrected data, leading to an error of 6.6.

The random α estimator hardly improves the performances, and its iterative variant even increases a little the error. Figure 16 shows that these methods lead to a better organization of the samples by drug, but still far from a clean clustering. The replicate-based method leads to better performances. Even though we do 200 runs of k-means to minimize the within sum of squares objective, different occurrences of the 200 runs lead to different clusterings with close objectives. We choose to indicate the range of clustering errors given by these different clusterings (2.8–3.8).

The iterative version of the estimator gives the same range of errors. Figure 17 shows that these corrections indeed lead to a better organization of the samples by drugs in the space spanned by the first two principal components, but fails to correct the time effect against which no replicate is available.

The deflation $\|W\alpha\|_F$ obtained by the naive RUV-2 and replicate based procedures are larger than the one obtained by the random α correction, but this is not the reason for the replicate based procedure to work better than the random α correction: the former is quite robust to changes in k and the latter does not improve when changing ν.

7. Benefit of control genes

In the experiments on gene expression data presented in Section 5 of the main manuscript, and Sections 5 and 6 of this supplementary material, we have assumed that control genes had little association with X and allowed proper estimation for the methods we introduced. In this section, we assess this hypothesis on our three benchmarks. Table 6 reproduces the results on the first two benchmarks of the non-iterative methods that we considered and which make use of control genes. In addition for each method and each of our first two benchmarks, we show the performance of

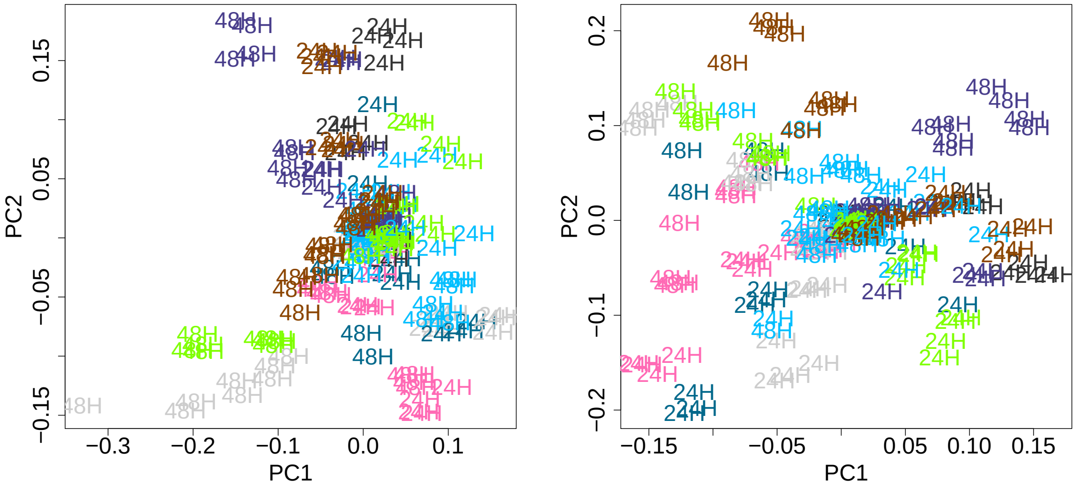

Fig. 16. Samples of the MAQC-II study represented in the space of their first two principal components after applying the random α correction (left panel) and its iterative variant (right panel). Each color represents a different drug. The labels indicate the time of each sample.

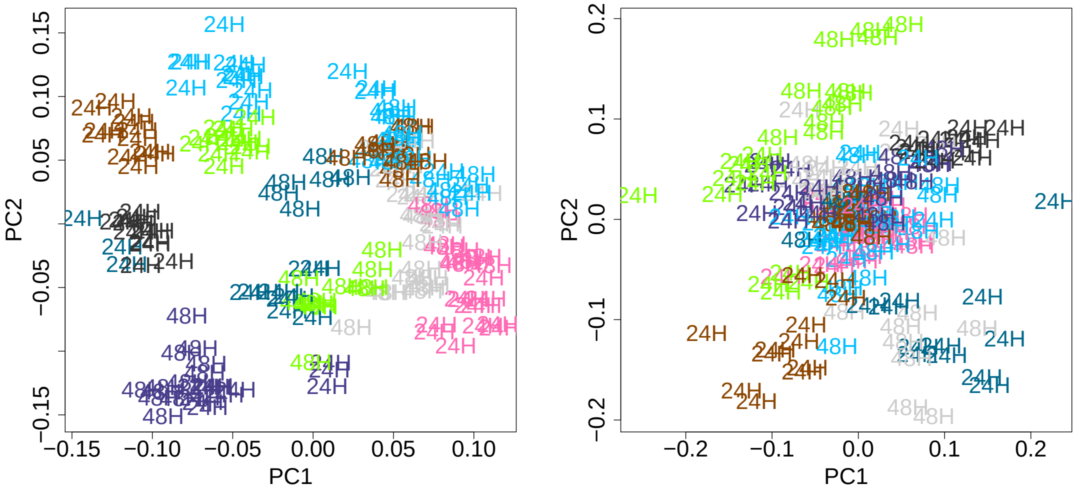

Fig. 17. Samples of the MAQC-II study represented in the space of their first two principal components after applying the replicate-based correction (left panel) and its iterative variant (right panel). Each color represents a different drug. The labels indicate the time of each sample.

the same method using all genes as control genes. For the gender data, we give the clustering error when filtering in 1260 genes, which correspond to the last point of Figure 3 in the main paper.

Method	Gender control	Gender all genes	GBM 1 control	GBM 1 all genes	GBM 2 control	GBM 2 all genes
Naive RUV-2	0.75	0.92	2	1.52	0	0.93
Replicate-based	0.77	0.77	0.2	0.25	0.61	0.37
Random α	0.43	0.99	0.21	0.24	1.5	1.8

Table 6. Clustering error of gender and glioblastoma data with full (1) or confounded (2) designs for various correction methods relying on control genes using either all genes or control genes.

The results of MAQC-II data are not presented in Table 6 but the result of each method is the same whether we use our control genes or all the genes for this dataset. Overall, we can see that some methods are affected by the use of control genes on the gender data, but using all the genes only mildly affects the performances of most methods on the GBM dataset, and as we said do not affect the performances on the MAQC-II dataset at all. This suggests that the genes that we used as control genes were indeed less affected by the factor of interest for the gender data but were not for the glioblastoma and MAQC-II data. This is consistent with the fact that methods which rely heavily on the control genes like naive RUV-2 and random α are very sensitive to the amplitude of the correction for the glioblastoma dataset and do not work for the MAQC-II dataset.

As one may expect from the discussion of Section 11 for this supplementary material, the replicate-based method introduced in Section 3 of the main manuscript is less affected than methods that rely solely on control genes, even on the gender dataset. Remember that our replicate-based procedure estimates W by regressing the control genes Y_c against the variations observed among contrasts of replicates which can make it robust to the fact that control genes are affected by the factor of interest.

In order to verify the fact that the control genes used for the gender data are good control

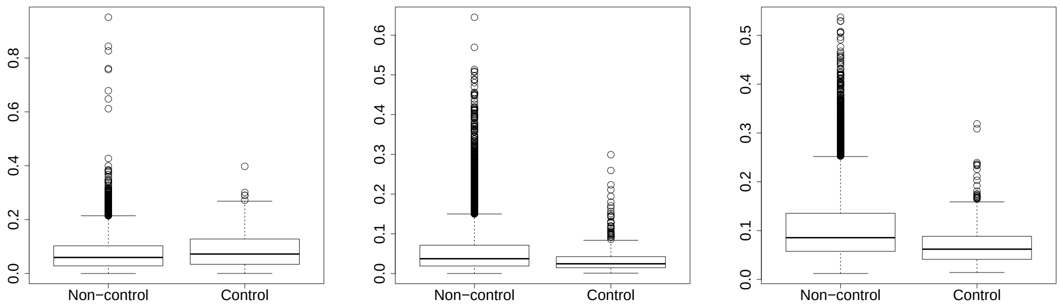

Fig. 18. boxplot of the CCA of control and non-control genes with the factor of interest X for the gender (left panel), glioblastoma (center panel) and MAQC-II (right panel) datasets.

genes whereas the ones used for the other datasets are not good control genes, we show the CCA of all control genes and all non-control genes with the factor of interest X as a boxplot for each dataset on Figure 18. Interestingly, the control genes used in the gender data are typically more associated with X than the non-control genes whereas the opposite is observed for the glioblastoma and MAQC-II datasets. This seems to contradict the fact that control genes help identifying W in the gender data and does not in the two others. Since W is essentially estimated using PCA on Y_c which is a multivariate procedure, we represent the first canonical correlation of X with the eigen space corresponding to the k first eigenvectors as a function of k on Figure 19. It is clear from the figure that for the gender dataset the eigen space built using control genes has a smaller association with X than the one built using non-control genes whereas this is much less clear for the two other datasets.

To conclude, the case of gender data suggests that when good control genes are available they do help estimate and remove unwanted variation, especially for estimators which do not use replicate samples. The notion of good control samples seems to have more to do with the fact that the directions of maximum variance among these genes are not associated with X than with individual univariate association of the genes with X. When control genes are as associated as the other genes with X, methods using replicate samples still give reasonable estimates and other

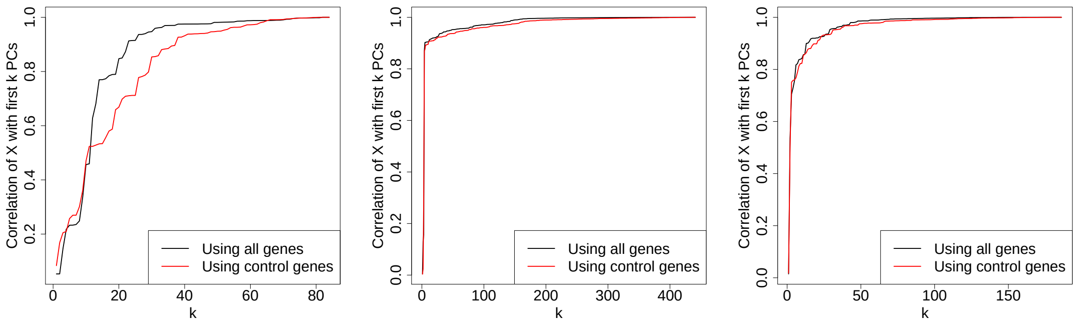

Fig. 19. First canonical correlation of the factor of interest X with the space spanned by the k first eigenvectors of the empirical covariance computed on control genes (in red) and non-control genes (in black) against k for the gender (left panel), glioblastoma (center panel) and MAQC-II (right panel) datasets.

methods become either ineffective or very sensitive to the amplitude of the correction.

8. Effect of hyperparameter misspecification for the experiments in Section 4

In Section 4 of the main manuscript, we used the same value of k (for naive RUV-2 and the replicate based method) and ν (for the random effect estimator) in the estimator and in the data generating model. More precisely for the random effect model, we use the known variance ratio $\nu_{pop} \triangleq \left(\sigma_\beta^2 + \sigma_\varepsilon^2\right)/\sigma_\alpha^2$ as ridge parameter for the random effect estimator: if there was no $X\beta$ term in the model, the ν such that $(W^\top W + \nu I_k)^{-1} W^\top Y$ is the maximum likelihood estimator of the RUV model would be $\sigma_\varepsilon^2/\sigma_\alpha^2$, but since the non-iterative random effect estimator does not account for the $X\beta$ term, the excess of variance must be counted in ν. In a sense, the non-iterative random effect estimator considers a modified ε which also contains $X\beta$. In addition for the random effect model and its iterative variants, we use $k = m$ instead of the true k to limit the number of hyperparameters.

We now consider the effect of using incorrect values for these hyperparameters.

Figure 20 shows the effect of misspecifying k on the naive RUV-2 estimator (3) for the three protocols — X independent of W, moderate association, $X = W$. The x-axis represents the k

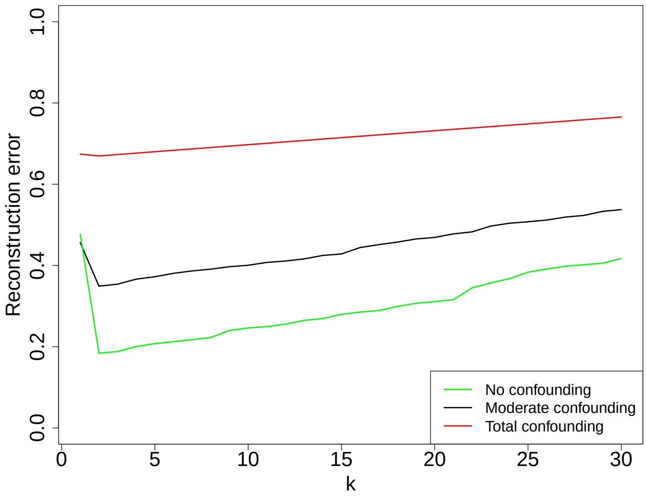

Fig. 20. Reconstruction error $\|(Y - \widehat{W\alpha}) - (Y - W\alpha)\|^2 / \|Y - W\alpha\|^2$ of naive RUV-2 as a function of the hyperparameter k for the three simulations.

used in the estimator, and the y-axis gives the corresponding reconstruction error. The correct $k = 2$ always performs best, and over or under estimation of k increases the error especially when X is different from W. When $X = W$, the reconstruction error starts high for $k = 2$ and is less affected by over estimation of k since most of the signal of interest is already removed at $k = 2$.

Figure 21 shows the effect of misspecifying ν on the random effect estimator (4). The x-axis represents the log ratio of the ν used for the estimator and the model ν: values of the ratio greater than 0 mean that we overestimated ν, values less than 0 mean that we underestimated it. As for k with the fixed effect method, misspecification of ν increases the reconstruction error, but the method shows some robustness to misspecification. The increase is slower in case of underestimation — removing too much signal — than overestimation — not removing enough.

Finally, Figure 22 represents the reconstruction error on the y-axis against the k used for the replicate-based estimator of $W\alpha$ introduced in Section 3 of the main manuscript. By construction, k cannot be larger than the rank of Y^d, which is 10 in this experiment. As we observed in Section 4 of the main manuscript, the method performs less well than control gene based correction in their

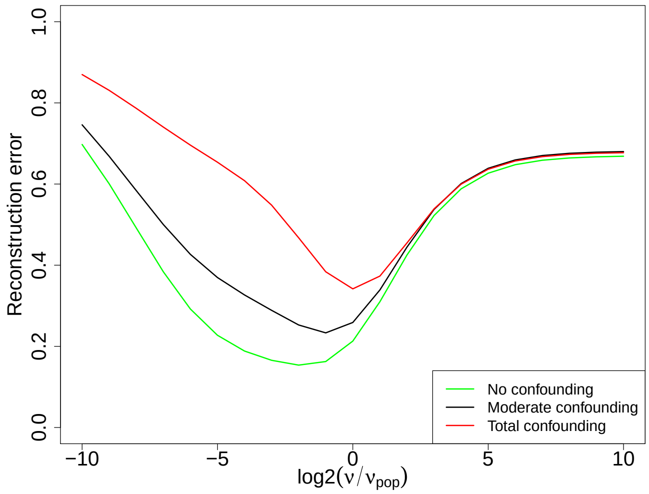

Fig. 21. Reconstruction error $\|(Y - \widehat{W\alpha}) - (Y - W\alpha)\|^2 / \|Y - W\alpha\|^2$ of the random α method as a function of the hyperparameter ν for the three simulations, using $k = m$.

ideal settings: when good control genes are used, in the presence of little confounding and using the correct hyperparameters. It is however essentially unaffected by the level of confounding of X and W and performs better than control-gene based methods in the presence of confounding or when the latter use incorrect values of k and ν.

Figure 22 suggest that the increase of reconstruction error caused by misspecification of k is similar to the one of control-gene based methods, but limited by construction since k cannot be larger than the rank of Y^d.

9. Effect of the unsupervised adjustment on differential analysis

The whole idea of removing unwanted variation without specifying a factor of interest can be confusing. Most methods in the literature on unwanted variation aim at getting a better power to detect differentially expressed genes with respect to a factor of interest, or at improving the prediction of this factor of interest.

By contrast, our objective is to correct the data when no factor of interest is specified, typically

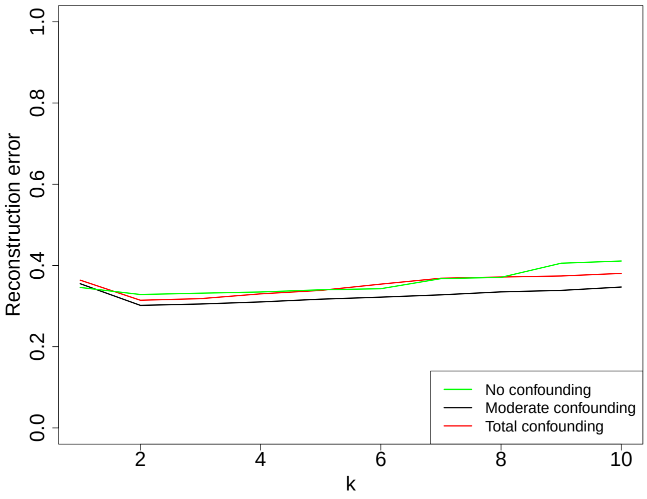

Fig. 22. Reconstruction error $\|(Y - \widehat{W\alpha}) - (Y - W\alpha)\|^2 / \|Y - W\alpha\|^2$ of the replicate-based method as a function of the hyperparameter k for the three simulations.

before conducting unsupervised analysis such as clustering or PCA. Testing for differential expression requires a factor of interest to be known, and as this factor becomes known, we recommend using targeted techniques, such as the ones introduced in Leek and Storey (2007); Listgarten *and others* (2010); Gagnon-Bartsch and Speed (2012); Gagnon-Bartsch *and others* (2013).

For the sake of completeness, we present here a few results obtained with the random α method (Section 2 of the main manuscript) on a differential analysis problem — which, again, would constitute a misuse in our view, and which we do not recommend. We use synthetic data, with the same setting as in Section 4 of the main manuscript, except that X has only one dimension and 90% of the β coefficients are 0. Accordingly, the baseline ν that we use for the random α technique is $0.9 * (\sigma_\varepsilon^2/\sigma_\alpha^2) + 0.1 * (\sigma_\varepsilon^2 + \sigma_\beta^2)/\sigma_\alpha^2)$. We also try strongly misspecified ν by multiplying this baseline by 0.01 and 100, and compare to uncorrected data, perfectly corrected data (using the true $W\alpha$ term) and the RUV-2 method of Gagnon-Bartsch and Speed (2012). RUV-2 uses the value of the factor of interest X to correct for unwanted variation and should not be confused with the naive RUV-2 technique used in the experiments of the main manuscript.

Plots on Figure 23 show the sensitivity vs specificity for t-tests in the three confounding settings used in Section 4 of the main manuscript: total confounding, moderate confounding and no confounding between the factor of interest X and the unwanted variation factor W. In the absence of confounding, the presence of unwanted variation has little effect on the power of the test and testing the uncorrected data (red line) yields a curve similar to that obtained when testing the perfectly corrected data (black line). The only approach behaving differently is the misspecified random α method with the 0.01 multiplier which removes too much variance and leads to a — limited — loss of power. Table 7 shows that the behavior in terms of adjustment — the actual purpose of our random α technique — is different: while leaving the unwanted variation in the data has little effect on the detection power, it yields a very different matrix than the one without unwanted variation, as reflected by the large reconstruction error. The random α method achieves a low error, which is then increased when ν is misspecified, but overcorrecting (factor 0.01) yields a much lower error than undercorrecting — the opposite behavior to what we observed on power.

In the case of a moderate confounding between X and W, not correcting the data yields a much lower power than perfectly correcting it. The random α method does not allow to recover all of the lost power, but leads to similar performances as RUV-2 – which uses the factor of interest. Hyperparameter misspecification lowers the performances: undercorrected data (factor 100) behave like uncorrected data, while overcorrected data have a larger power.

Finally in the total confounding setting, the presence of unwanted variation causes a large loss of power. The random α method leads to little improvement. Undecorrected data behave like uncorrected data, and overcorrected data lead to almost no power. Here again, we observe the opposite behavior in terms of reconstruction error: overcorrecting gives a much lower error than undercorrecting – and both yield a lower error than the uncorrected data. Interestingly, RUV-2 which is targeted to differential analysis also loses its power: when $X = W$, its fixed effect

	Uncorrected	random α	$\nu \times 0.01$	$\nu \times 100$	RUV-2
Independent	1.7	0.08	0.62	1.4	0.04
Moderate	1.7	0.15	0.65	1.44	0.12
Confounded	1.7	0.18	0.67	1.47	3.36

Table 7. Reconstruction errors $\|(Y - \widehat{W\alpha}) - (Y - W\alpha)\|^2 / \|Y - W\alpha\|^2$ for the synthetic data used in Figure 23.

model becomes unidentifiable. In Gagnon-Bartsch *and others* (2013), we discuss random effect models targeted to differential analysis, which use X and would be able to deal better with this somewhat degenerate setting.

To conclude, we observe some level of association between the quality of adjustment — which is the objective of our methods — and power for testing the effect of the factor of interest: very good reconstructions generally yield more power than poor reconstructions. However this association is not direct and reliable: sometimes a method which better removes unwanted variation performs less well at testing. This is consistent with what we observe in Table 1, 2 and 3, where some methods lead to lower clustering errors but detect fewer genes on the sex chromosomes. We emphasize that when the objective is to apply hypothesis testing procedures, more targeted techniques than the ones introduced in this work should be used.

10. Using $\hat{W}_r$ using all genes as control genes

The extreme case where all genes are used as control genes is of interest. In this case $\hat{W}_r \hat{\alpha} = Y \hat{\alpha}_c^\top (\hat{\alpha}_c \hat{\alpha}_c^\top)^{-1} \hat{\alpha} = Y Q_k Q_k^\top$, where Q_k are the first k right singular vectors of Y^d. Finally, $Y - \hat{W}_r \hat{\alpha} = Y(I - Q_k Q_k^\top)$: using all genes as control genes amounts to projecting the samples onto the orthogonal complement to the span of the first k right singular vectors of Y^d. This shows how the replicate-based correction is dual to naive RUV-2: it uses negative control samples rather than genes, and amounts to projecting samples, rather than genes, to some subspace. As for the effect of the correction, assuming $\hat{\alpha} \approx \alpha$, and the RUV model of the main manuscript, it holds asymptotically that $\hat{W}_r \hat{\alpha} = Y \hat{\alpha}^\top (\hat{\alpha} \hat{\alpha}^\top)^{-1} \hat{\alpha} \approx W\alpha$, since dot products between independent

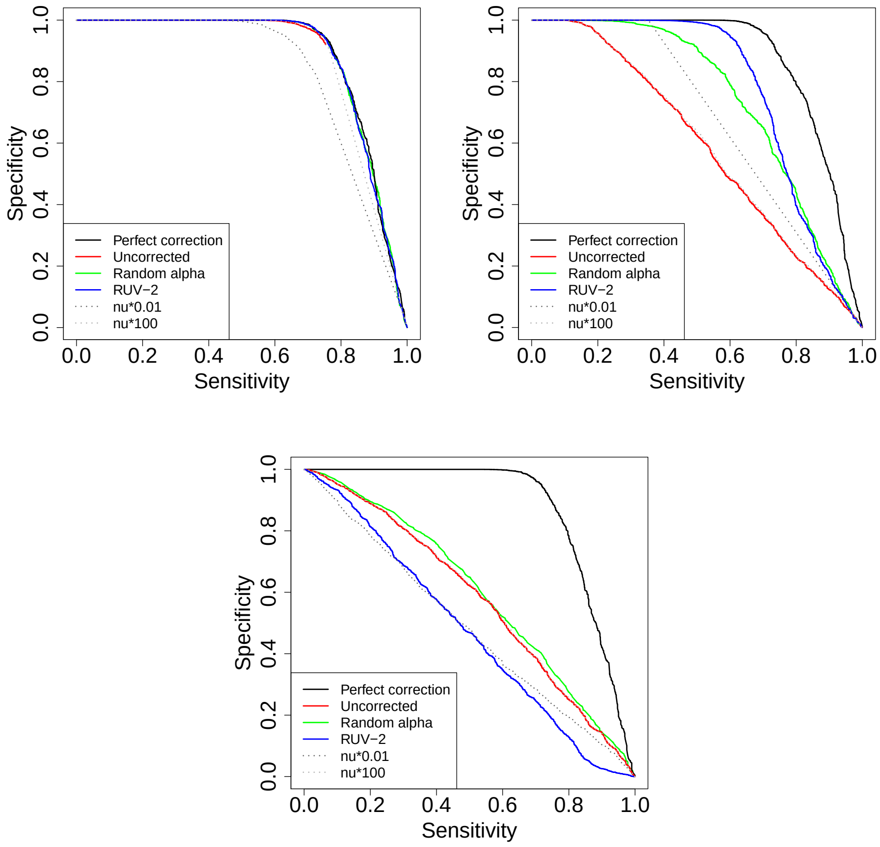

Fig. 23. Sensitivity vs specificity after various correction techniques with no (top left), partial (top right) and total confounding (bottom).

multivariate normal variables are close to 0 in high dimension. This is a random effect point of view, the fixed effect version being that $\hat{W}_r\hat{\alpha} \approx W\alpha$ if $\hat{\alpha} \approx \alpha$ and $\beta\alpha^\top(\alpha\alpha^\top)^{-1}\alpha$ has a small norm, which corresponds to the condition on the correlation between W and X for naive RUV-2 to work. In practice, this different condition means that $\hat{W}_r\hat{\alpha}$ can work well even if there is a high level of confounding between X and W, but requires that their respective effects β and α be uncorrelated — a similar discussion is provided in Section 3.6.3 of Gagnon-Bartsch *and others* (2013) for the RUV-4 technique when X is observed. This analysis suggests that even without negative control genes, the replicate-based method may still provide useful corrections.

11. Comparison of the two estimators of W

The procedure described in Section 3 of the main manuscript yields an estimator of W, which can be plugged in any of the procedures we discussed in Section 2 of the main manuscript. The estimator $\hat{W}_2$ we considered so far was obtained using the first left singular vectors of the control genes Y_c, which can also be thought of as a regression of the control genes on their first right singular vectors, *i.e.*, the main variations $E_k Q^\top$ observed in the control genes. By contrast the estimator $\hat{W}_r$ introduced in Section 3 of the main manuscript is obtained by a regression of the control genes against the main variations observed in the control genes for the control samples formed by differences of replicates.

Assuming our control genes are influenced by the factor of interest X, *i.e.*, $\beta_c \neq 0$, the estimator of W based solely on control genes may have more association with X than it should, whereas the one using differences of replicate samples should not be affected. On the other hand, restricting ourselves to the variation observed in differences of replicates may be too restrictive because we don't capture unwanted variation when no replicates are available.

To make things more precise, let us assume that the control genes are actually influenced by the factor of interest X and that $\beta_c \sim \mathcal{N}(0, \sigma^2_{\beta_c})$. In this case we have $\mathbb{E}[Y_c Y_c^\top] = XX^\top \sigma^2_{\beta_c} + WW^\top \sigma^2_\alpha + I_m \sigma^2_\varepsilon$, so if we use Y_c to estimate W or Σ as we do for $\hat{W}_2$ the estimate will be biased towards X.

Let us now consider the estimator $\hat{W}_r$ obtained by the replicate based procedure. To simplify the analysis we assume that $k = d$ and therefore $\hat{\alpha} = Y^d$ in the procedure described in Section 3 of the main manuscript. Consequently $\hat{W}\hat{\alpha} = Y_c (Y_c^d)^\top \left(Y_c^d (Y_c^d)^\top\right)^{-1} Y^d$. Define $\hat{W}_r \triangleq Y_c (Y_c^d)^\top \left(Y_c^d (Y_c^d)^\top\right)^{-\frac{1}{2}}$. Assuming X^d is indeed equal to 0 we can develop:

$$
\begin{aligned}
\hat{W}_r =& (X\beta_c + W\alpha_c + \varepsilon_c)(W^d \alpha_c + \varepsilon_c^d)^\top \\
& \left(W^d \alpha_c \alpha_c^\top (W^d)^\top + W^d \alpha_c (\varepsilon_c^d)^\top + \varepsilon_c^d \alpha_c^\top (W^d)^\top + \varepsilon_c^d (\varepsilon_c^d)^\top\right)^{-\frac{1}{2}}.
\end{aligned}
$$

We now make some heuristic asymptotic approximations in order to get a sense of the behavior of $\hat{W}_r$. $\alpha_c\alpha_c^\top$ and $\varepsilon_c^d(\varepsilon_c^d)^\top$ are Wishart variables which by the central limit theorem are close to $cI_m\sigma_\alpha^2$ and $cI_m\sigma_\varepsilon^2$ respectively if the number c of control genes is large enough regardless how good the control genes are, *i.e.*, how small σ_{β_c} is. In addition dot products between independent multivariate normal variables are close to 0 in high dimension so we approximate $\beta_c\alpha_c^\top, \beta_c\varepsilon_c^\top$ and $\alpha_c\varepsilon_c^\top$ by 0. The approximations involving β_c depend in part how good the control genes are, but can still be valid for larger σ_{β_c} if the number of control genes is large enough. We further assume that $\sigma_\varepsilon \ll \sigma_\alpha$ and that the control samples are independent from the samples for which we estimate W and ignore the $cI_m\sigma_\varepsilon^2$ and $\varepsilon_c(\varepsilon_c^d)^\top$ terms.

Implementing all these approximations yields $\hat{W}_r \simeq \sigma_\alpha c^{\frac{1}{2}} W(W^d)^\top \left(W^d(W^d)^\top\right)^{-\frac{1}{2}}$. Writing $W^d = A\Delta B^\top$ for the SVD of W^d, we obtain $\hat{W}_r \simeq \sigma_\alpha c^{\frac{1}{2}} W B_r A_r^\top$, where r is the rank of W^d and A_r, B_r contain the first r columns of A and B respectively. First it is interesting to note that this approximation does not depend on the control genes anymore. In the experiments on synthetic data of Section 4 of the main manuscript, using lots of non-control genes yields better estimates than a few control genes.

This suggests moreover that if W^d has rank k $\hat{W}_r$ is a good estimator of W in the sense that it is not biased towards X even if the control genes are influenced by X. If W^d is column rank deficient, the B_r mapping can delete or collapse unwanted factors in $\hat{W}_r$.

The effect is easier to observe on the estimator of the covariance Σ of the residuals $Y - X\beta$: $\hat{W}_r\hat{W}_r^\top \simeq \sigma_\alpha^2 c W B_r B_r^\top W^\top$. Consider for example the following case with 3 unwanted factors and 3 replicate samples with unwanted variation $(1,0,3)$, $(0,1,3)$ and $(1,1,3)$. The W^d and corresponding $B_rB_r^\top$ obtained by taking differences between replicates $(1,2)$, $(1,3)$ and $(3,2)$ are

$$W^d = \begin{pmatrix} 1 & -1 & 0 \\ 0 & -1 & 0 \\ 1 & 0 & 0 \end{pmatrix} \quad \text{and} \quad B_rB_r^\top = \begin{pmatrix} 1 & 0 & 0 \\ 0 & 1 & 0 \\ 0 & 0 & 0 \end{pmatrix},$$

so the $B_rB_r^\top$ factor removes the third factor from the estimate of Σ. This is because the 3 replicates have the same value for the third factor.

Similarly if two factors are perfectly correlated on the replicate samples, *e.g.*, the first two factors for $(1,1,0)$, $(0,0,1)$ and $(1,1,1)$, the W^d and corresponding $B_r B_r^\top$ for the same differences between replicates $(1,2)$, $(1,3)$ and $(3,2)$ are

$$W^d = \begin{pmatrix} 1 & 1 & -1 \\ 0 & 0 & -1 \\ 1 & 1 & 0 \end{pmatrix} \qquad \text{and} \qquad B_r B_r^\top = \begin{pmatrix} 1/2 & 1/2 & 0 \\ 1/2 & 1/2 & 0 \\ 0 & 0 & 1 \end{pmatrix},$$

which collapses the first two factors into an average factor and leaves the third one unchanged.

Finally, another option in the context of random α models is to combine the control gene based and replicate based estimators of W by concatenating them. In terms of Σ, this amounts to summing the two estimators of the covariance matrix. This may help if, as in our first example, some factors are missing from $\hat{W}_r$ because all pairs of replicates have the same value for these factors. In this case, combining it with $\hat{W}_2$ could lead two an estimate containing less X but still containing all the unwanted factors.

12. Alternative formulation of the random effect estimator

The following proposition provides an alternative formulation of the random effect estimator introduced in the main manuscript:

Proposition 1 Let $R \in \mathbb{R}^{m \times n}$, $W \in \mathbb{R}^{m \times k}$, $\nu > 0$, then

$$\min_{\alpha \in \mathbb{R}^{k \times n}} \left\{ \|R - W\alpha\|_F^2 + \nu \|\alpha\|_F^2 \right\} = \|R\|_{S(W,\nu)}^2, \tag{12.1}$$

where $S(W,\nu) \triangleq \nu^{-1} \left(WW^\top + \nu I_m \right)$.

Proof. The left hand side of (12.1) is a standard ridge regression and has a closed form solution:

$$\alpha^* \triangleq \arg\min_{\alpha \in \mathbb{R}^{k \times n}} \left\{ \|R - W\alpha\|_F^2 + \nu \|\alpha\|_F^2 \right\} = \left(W^\top W + \nu I_k \right)^{-1} W^\top R,$$

so for any $R \in \mathbb{R}^{k \times n}$,

$$\min_{\alpha\in\mathbb{R}^{k\times n}}\left\{\|R-W\alpha\|_F^2+\nu\|\alpha\|_F^2\right\}=\left\|\left(I_m-W\left(W^\top W+\nu I_k\right)^{-1}W^\top\right)R\right\|_F^2$$
$$+\nu\left\|\left(W^\top W+\nu I_k\right)^{-1}W^\top R\right\|_F^2$$
$$=\mathbf{tr}\ R^\top\Big(I_m-2W\left(W^\top W+\nu I_k\right)^{-1}W^\top+W\left(W^\top W+\nu I_k\right)^{-1}W^\top W\left(W^\top W+\nu I_k\right)^{-1}W^\top$$
$$+\nu W\left(W^\top W+\nu I_k\right)^{-2}W^\top\Big)R,$$

where we used the fact that $\|A\|_F^2=\mathbf{tr}\ A^\top A$. This is $\mathbf{tr}\ R^\top\left(I_m+WBW^\top\right)R$ with:

$$B\triangleq\left(W^\top W+\nu I_k\right)^{-1}W^\top W\left(W^\top W+\nu I_k\right)^{-1}+\nu\left(W^\top W+\nu I_k\right)^{-2}-2\left(W^\top W+\nu I_k\right)^{-1}$$
$$=\left(W^\top W+\nu I_k\right)^{-1}\left(W^\top W\left(W^\top W+\nu I_k\right)^{-1}+\nu\left(W^\top W+\nu I_k\right)^{-1}-2I_k\right)$$
$$=\left(W^\top W+\nu I_k\right)^{-1}\left(\left(W^\top W+\nu I_k\right)\left(W^\top W+\nu I_k\right)^{-1}-2I_k\right)$$
$$=-\left(W^\top W+\nu I_k\right)^{-1},$$

so

$$\min_{\alpha\in\mathbb{R}^{k\times n}}\left\{\|R-W\alpha\|_F^2+\nu\|\alpha\|_F^2\right\}=\mathbf{tr}\ R^\top\left(I_m-W\left(W^\top W+\nu I_k\right)^{-1}W^\top\right)R$$
$$=\|R\|_{S(W,\nu)}^2.$$

□

$$\min_{X\beta\in\mathcal{M}}\|Y-X\beta\|_{(WW^\top+\nu I_m)}^2,\tag{12.2}$$

is the maximum likelihood estimator of $X\beta$ for the model $Y=X\beta+\eta$, where $\eta_j\sim\mathcal{N}(0,WW^\top+\nu I_m)$, $j=1,\ldots,n$, equivalent to the random α model introduced in the main manuscript. $\|Y-X\beta\|_{(WW^\top+\nu I_m)}^2$ generalizes the regular ℓ_2 objective of classical unsupervised problem such as k-means and PCA to include a covariance information over the observations. If the objective is to estimate $X\beta$, *e.g.*, to do clustering, one could directly try to minimize $\|Y-X\beta\|_{(WW^\top+\nu I_m)}^2$.

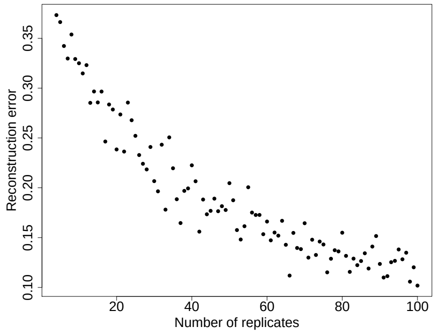

Fig. 24. Reconstruction error $\|(Y - \widehat{W\alpha}) - (Y - W\alpha)\|^2 / \|Y - W\alpha\|^2$ of the replicate based method as a function of the number of replicates in the independent setting.

While both formulations have the same set of global minimizers $X\beta$, neither of the formulations can be solved exactly in general, and for some $\mathcal{M}$ the global minimizers of one may be better estimators than those of the other. We tried such an approach for k-means clustering with no success.

13. Effect of the number of replicate samples on the performance of the replicate-based method

Figure 24 shows the reconstruction error as a function of the number of replicates in the independent setting of the experiments on synthetic data (first column of Table 1). It suggests that a large number of replicates — around 30/100 here — could be required to match the performance obtained with the random effect estimators using good control genes. It is however a safer alternative against confounding or poor quality of control genes.

14. Correlation on samples vs correlation on genes

In model (4) we arbitrarily chose to endow α with a distribution, which is equivalent to introducing an $m \times m$ covariance matrix Σ on the rows of the $Y - X\beta$ residuals. If we choose instead to model

the rows of W as iid normal vectors with spherical covariance, we introduce a $n \times n$ covariance matrix Σ' on the columns of the $Y - X\beta$ residuals. If X is observed and if we consider a random β as well, the maximum a posteriori estimator of β incorporates the prior encoded in Σ' by shrinking the β_j of positively correlated genes towards the same value and enforcing a separation between the β_j of negatively correlated genes. This approach was used in Desai and Storey (2012). As an example if $\alpha \in \mathbb{R}^{1\times n}$ is a constant vector, *i.e.*, if Σ' is a constant matrix with an additional positive value on its diagonal, the maximum a posteriori estimator of β boils down to the "multi-task learning" estimator (Evgeniou *and others*, 2005; Jacob *and others*, 2009) detailed in Section 15 of this supplementary material. This model as is does not deal with any source of unwanted variation which may affect the samples. Using two noise terms in the regression model, one with correlation on the samples and the other one on the genes would lead to the same multi-task penalty shrinking some β_j together, but with a $\|.\|^2_\Sigma$ loss instead of the regular Frobenius loss discussed in Section 15 of this supplementary material.

This discussion assumes Σ' is known and used to encode some prior on the residuals of the columns of $Y - X\beta$. If however Σ' needs to be estimated, and estimation is done using the empirical covariance of $Y - X\beta$, the estimators of β derived from (4) and from the model with an $n \times n$ covariance Σ' on the $Y - X\beta$ residuals become very similar, the only difference being that in one case the estimator of $\alpha|W, Y - X\beta$ is shrinked and in the other case the estimator of $W|\alpha, Y - X\beta$ is shrinked.

15. Estimator of β for a particular Σ' defined on the columns of the residuals

In this Section, we show how a model similar to (4) and discussed in Section 14 of this supplementary material is related to a "multi-task learning" estimator (Evgeniou *and others*, 2005; Jacob *and others*, 2009).

We consider the following model:

$$Y = X\beta + \tilde{\varepsilon}, \tag{15.3}$$

where $Y, \tilde{\varepsilon} \in \mathbb{R}^{m\times n}$, $X \in \mathbb{R}^{m\times p}$, $\beta \in \mathbb{R}^{p\times n}$. We further assume that each row of $\tilde{\varepsilon}$ is distributed as $\mathcal{N}(0, \Sigma')$ where $\Sigma' \in \mathbb{R}^{n\times n}$ is a covariance matrix. This is different from (4) where the $m \times m$ covariance was defined on the rows of $\tilde{\varepsilon}$.

(15.3) is equivalent to

$$Y = X\beta + W\alpha + \varepsilon, \tag{15.4}$$

where each column W_j of W is such that $W_j \overset{iid}{\sim} \mathcal{N}(0, \sigma_W^2 I_m)$, $\varepsilon_{ij} \overset{iid}{\sim} \mathcal{N}(0, \sigma^2)$ and $\alpha \in \mathbb{R}^{k\times n}$ for some $k \leqslant m$ is such that $\alpha^\top\alpha + \sigma^2 I_n = \Sigma'$.

Assume $k = 1$ and $\alpha = \mathbf{1}^\top$, where $\mathbf{1}$ is the all-one vector in $\mathbb{R}^n$. Then $\Sigma' = \sigma^2 I_n + \mathbf{1}\mathbf{1}^\top$, *i.e.* a constant matrix plus some additional constant on the diagonal. In this special case, W is a single standard normal column vector and (15.4) can be written:

$$\begin{aligned} Y &= X\beta + W\alpha + \varepsilon \\ &= X\beta + W\mathbf{1}^\top + \varepsilon \\ &= X\left(\beta + (X^\top X)^{-1}X^\top W\mathbf{1}^\top\right) + \varepsilon + R, \end{aligned}$$

where R is the projection of $W\mathbf{1}^\top$ to the orthogonal space of X. We can disregard it because a noise orthogonal to X has no effect on a regression against X. Denoting $V \triangleq (X^\top X)^{-1}X^\top W\mathbf{1}^\top$, we see that (15.3) with this particular covariance is equivalent to assuming $Y = Xb + \varepsilon$, where $b = \beta + V$. Each column of V is equal to $v = (X^\top X)^{-1}X^\top W$, *i.e.*, to the projection of W on X. If β is assumed to be non-random and we estimate it by maximum likelihood we recover the regular OLS: $\beta + V$ is not identifiable. If we add a normal prior on β, the maximum a posteriori

(MAP) equation is:

$$\max_{\beta,v} L(\beta, v|X, Y) \propto \max_{\beta,v} L(X, Y|\beta, v)p(\beta)p(v) \tag{15.5}$$

$$= \max_{\beta,v} \{\log L(X, Y|\beta, v) + \log p(\beta) + \log p(v)\} \tag{15.6}$$

$$= \min_{\beta,v} \|Y - X(\beta + V)\|_F^2 + \lambda\|\beta\|_F^2 + \nu\|v\|_F^2, \tag{15.7}$$

where λ, ν depend on the prior variances of β and α. Then plugging $b = \beta + V$ and denoting its columns by b_i,

$$\begin{aligned}
&\min_{\beta,v} \left\{\|Y - X(\beta + V)\|_F^2 + \lambda\|\beta\|_F^2 + \nu\|v\|_F^2\right\} \\
&= \min_{b,v} \left\{\|Y - Xb\|_F^2 + \lambda \sum_{i=1}^{n} \|b_i - v\|_F^2 + \nu\|v\|_F^2\right\} \\
&= \min_{b} \left\{\|Y - Xb\|_F^2 + \lambda \min_{v} \left(\sum_{i=1}^{n} \|b_i - v\|_F^2 + \frac{\nu}{\lambda}\|v\|_F^2\right)\right\} \\
&= \min_{b} \left\{\|Y - Xb\|_F^2 + \lambda \sum_{i=1}^{n} \|b_i - \bar{b}\|_F^2 + \nu\|\bar{b}\|_F^2\right\},
\end{aligned}$$

where $\bar{b} \triangleq \arg\min_v \left(\sum_{i=1}^{n} \|b_i - v\|_F^2 + \frac{\nu}{\lambda}\|v\|_F^2\right)$ is a shrinked average of the b_i. The first equality is replacing $\beta + V$ by b and β by $b - V$. The second equality is moving the $\min_v$ to the part which depends on v. The last equality carries out the minimization over v.

Adding a block structure to Σ', or equivalently rows to α which are 1 for some genes and 0 for others, leads to an additional regularizer which penalizes the sum of squares among the β_i within each block.

References

Alter, O., Brown, P. O. and Botstein, D. (2000, Aug). Singular value decomposition for genome-wide expression data processing and modeling. *PNAS* **97**(18), 10101–10106.

Benito, Monica, Parker, Joel, Du, Quan, Wu, Junyuan, Xiang, Dong, Perou,

Charles M and Marron, J. S. (2004, Jan). Adjustment of systematic microarray data biases. *Bioinformatics* **20**(1), 105–14.

Cancer Genome Atlas Research Network. (2008, Oct). Comprehensive genomic characterization defines human glioblastoma genes and core pathways. *Nature* **455**(7216), 1061–1068.

Desai, Keysur H and Storey, John D. (2012). Cross-dimensional inference of dependent high-dimensional data. *Journal of the American Statistical Association* **107**(497), 135–151.

Evgeniou, T., Micchelli, C. and Pontil, M. (2005). Learning multiple tasks with kernel methods. *J. Mach. Learn. Res.* **6**, 615–637.

Freedman, D. (2005). *Statistical Models: Theory And Practice*. Cambridge University Press.

Gagnon-Bartsch, Johann, Jacob, Laurent and Speed, Terence P. (2013). Removing unwanted variation from high dimensional data with negative controls. *Technical Report*, UC Berkeley. Technical report 820. Monograph in preparation.

Gagnon-Bartsch, Johann A. and Speed, Terence P. (2012, Jul). Using control genes to correct for unwanted variation in microarray data. *Biostatistics* **13**(3), 539–552.

Hotelling, H. (1936). Relation between two sets of variates. *Biometrika* **28**, 322–377.

Hyvrinen, A and Oja, E. (2000). Independent component analysis: algorithms and applications. *Neural Networks: The Official Journal of the International Neural Network Society* **13**(4-5), 411–430. PMID: 10946390.

Jacob, L., Bach, F. and Vert, J.-P. (2009). Clustered multi-task learning: A convex formulation. In: *Advances in Neural Information Processing Systems 21*. MIT Press, pp. 745–752.

Johnson, W. Evan, Li, Cheng, Biostatistics, Department, Biology, Computational and Rabinovic, Ariel. (2007). Adjusting batch effects in microarray expression data using empirical bayes methods. *Biostatistics* **1**(8), 118–127.

Kang, Hyun Min, Ye, Chun and Eskin, Eleazar. (2008, Dec). Accurate discovery of expression quantitative trait loci under confounding from spurious and genuine regulatory hotspots. *Genetics* **180**(4), 1909–1925.

Leek, Jeffrey T and Storey, John D. (2007, Sep). Capturing heterogeneity in gene expression studies by surrogate variable analysis. *PLoS Genet* **3**(9), 1724–1735.

Li, Cheng and Wong, Wing Hung. (2003). *The analysis of gene expression data: methods and software*, Chapter DNA-Chip Analyzer (dChip). Springer, New York, pp. 120–141.

Listgarten, Jennifer, Kadie, Carl, Schadt, Eric E and Heckerman, David. (2010, Sep). Correction for hidden confounders in the genetic analysis of gene expression. *Proc Natl Acad Sci U S A* **107**(38), 16465–16470.

Luo, J. *and others*. (2010, August). A comparison of batch effect removal methods for enhancement of prediction performance using MAQC-II microarray gene expression data. *The pharmacogenomics journal* **10**(4), 278–291.

Marron, J. S., Todd, M. and Ahn, J. (2007). Distance weighted discrimination. *Journal of the American Statistical Association* **102**, 1267–1271.

Nielsen, Torsten O, West, Rob B, Linn, Sabine C, Alter, Orly, Knowling, Margaret A, O'Connell, John X, Zhu, Shirley, Fero, Mike, Sherlock, Gavin, Pollack, Jonathan R, Brown, Patrick O, Botstein, David *and others*. (2002, Apr). Molecular characterisation of soft tissue tumours: a gene expression study. *Lancet* **359**(9314), 1301–1307.

Prechelt, Lutz. (1997). Early stopping - but when? In: *Neural Networks: Tricks of the Trade, volume 1524 of LNCS, chapter 2*. Springer-Verlag. pp. 55–69.

Price, Alkes L, Patterson, Nick J, Plenge, Robert M, Weinblatt, Michael E, Shadick, Nancy A and Reich, David. (2006, Aug). Principal components analysis corrects for stratification in genome-wide association studies. *Nat Genet* **38**(8), 904–909.

Rhodes, Daniel R, Kalyana-Sundaram, Shanker, Mahavisno, Vasudeva, Varambally, Radhika, Yu, Jianjun, Briggs, Benjamin B, Barrette, Terrence R, Anstet, Matthew J, Kincead-Beal, Colleen, Kulkarni, Prakash, Varambally, Sooryanaryana, Ghosh, Debashis *and others*. (2007, Feb). Oncomine 3.0: genes, pathways, and networks in a collection of 18,000 cancer gene expression profiles. *Neoplasia* **9**(2), 166–180.

Rhodes, Daniel R, Yu, Jianjun, Shanker, K., Deshpande, Nandan, Varambally, Radhika, Ghosh, Debashis, Barrette, Terrence, Pandey, Akhilesh and Chinnaiyan, Arul M. (2004). Oncomine: a cancer microarray database and integrated data-mining platform. *Neoplasia* **6**(1), 1–6.

Shi, Leming *and others*. (2010, Aug). The microarray quality control (maqc)-ii study of common practices for the development and validation of microarray-based predictive models. *Nat Biotechnol* **28**(8), 827–838.

Sun, Yunting, Zhang, Nancy and Owen, Art. (2012). Multiple hypothesis testing adjusted for latent variables, with an application to the agemap gene expression data. *The Annals of Applied Statistics* **6**(4), 1349–1997.

Teschendorff, Andrew E., Zhuang, Joanna and Widschwendter, Martin. (2011). Independent surrogate variable analysis to deconvolve confounding factors in large-scale microarray profiling studies. *Bioinformatics* **27**(11), 1496–1505.

Verhaak, Roel G W *and others*. (2010, Jan). Integrated genomic analysis identifies clinically relevant subtypes of glioblastoma characterized by abnormalities in pdgfra, idh1, egfr, and nf1. *Cancer Cell* **17**(1), 98–110.

Walker, Wynn L, Liao, Isaac H, Gilbert, Donald L, Wong, Brenda, Pollard, Katherine S, McCulloch, Charles E, Lit, Lisa and Sharp, Frank R. (2008). Empirical bayes accomodation of batch-effects in microarray data using identical replicate reference samples: application to rna expression profiling of blood from duchenne muscular dystrophy patients. *BMC Genomics* **9**, 494.

Yang, Can, Wang, Lin, Zhang, Shuqin and Zhao, Hongyu. (2013, Apr). Accounting for non-genetic factors by low-rank representation and sparse regression for eqtl mapping. *Bioinformatics* **29**(8), 1026–1034.
